# Phenotypic dynamics and temporal heritability of tomato architectural traits using an unmanned ground vehicle-based plant phenotyping system

**DOI:** 10.1093/hr/uhaf109

**Published:** 2025-04-30

**Authors:** Pengyao Xie, Xin Yang, Leisen Fang, Tonglin Wang, Jirong Zheng, Yu Jiang, Haiyan Cen

**Affiliations:** College of Biosystems Engineering and Food Science, Zhejiang University, 866 Yuhangtang Road, Xihu District, Hangzhou 310058, China; Key Laboratory of Spectroscopy Sensing, Ministry of Agriculture and Rural Affairs, Zhejiang University, 866 Yuhangtang Road, Xihu District, Hangzhou 310058, China; College of Biosystems Engineering and Food Science, Zhejiang University, 866 Yuhangtang Road, Xihu District, Hangzhou 310058, China; Key Laboratory of Spectroscopy Sensing, Ministry of Agriculture and Rural Affairs, Zhejiang University, 866 Yuhangtang Road, Xihu District, Hangzhou 310058, China; College of Biosystems Engineering and Food Science, Zhejiang University, 866 Yuhangtang Road, Xihu District, Hangzhou 310058, China; Key Laboratory of Spectroscopy Sensing, Ministry of Agriculture and Rural Affairs, Zhejiang University, 866 Yuhangtang Road, Xihu District, Hangzhou 310058, China; Hangzhou Academy of Agricultural Sciences, 261 Zhusi Road, Xihu District, Hangzhou 310024, China; Hangzhou Academy of Agricultural Sciences, 261 Zhusi Road, Xihu District, Hangzhou 310024, China; Horticulture Section, School of Integrative Plant Science, Cornell University, 635 West North Street, Geneva, NY 14456, USA; College of Biosystems Engineering and Food Science, Zhejiang University, 866 Yuhangtang Road, Xihu District, Hangzhou 310058, China; Key Laboratory of Spectroscopy Sensing, Ministry of Agriculture and Rural Affairs, Zhejiang University, 866 Yuhangtang Road, Xihu District, Hangzhou 310058, China

## Abstract

Large-scale manual measurements of plant architectural traits in tomato growth are laborious and subjective, hindering deeper understanding of temporal variations in gene expression heterogeneity. This study develops a high-throughput approach for characterizing tomato architectural traits at different growth stages and mapping temporal broad-sense heritability using an unmanned ground vehicle-based plant phenotyping system. The SegFormer with fusion of multispectral and depth imaging modalities was employed to semantically segment plant organs from the registered RGB-D and multispectral images. Organ point clouds were then generated and clustered into instances. Finally, six key architectural traits, including fruit spacing (FS), inflorescence height (IH), stem thickness (ST), leaf spacing (LS), total leaf area (TLA), and leaf inclination angle (LIA) were extracted and the temporal broad-sense heritability folds were plotted. The root mean square errors (RMSEs) of the estimated FS, IH, ST, and LS were 0.014, 0.043, 0.003, and 0.015 m, respectively. The visualizations of the estimated TLA and LIA matched the actual growth trends. The broad-sense heritability of the extracted traits exhibited different trends across the growth stages: (i) ST, IH, and FS had a gradually increased broad-sense heritability over time, (ii) LS and LIA had a decreasing trend, and (iii) TLA showed fluctuations (i.e. an M-shaped pattern) of the broad-sense heritability throughout the growth period. The developed system and analytical approach are promising tools for accurate and rapid characterization of spatiotemporal changes of tomato plant architecture in controlled environments, laying the foundation for efficient crop breeding and precision production management in the future.

## Introduction

Tomatoes, with their unique flavor and high nutritional value, are greenhouse cash crops widely cultivated around the world. Despite significant advances in breeding programs aimed at improving yield and quality, considerable effort is still required to bridge the gap between the ever-increasing consumer demand and tomato production [1]. Research on tomato architecture is crucial because the ideotype exhibits favorable phenological characteristics, excellent light interception and photosynthetic capacity, and optimal nutrient allocation, all of which are essential for tomato growth and yield [2–5]. Several key traits such as stem thickness (ST), leaf spacing (LS), inflorescence height (IH), fruit spacing (FS), total leaf area (TLA), and leaf inclination angle (LIA) are considered as the fundamental traits of tomato architecture. ST reflects the vigor and robustness of the plant [6], LS determines the compactness of the plant architecture [7, 8], IH signifies the earliness of tomato ripening [9, 10], and FS represents the fruit yield per unit height [11]. TLA and LIA are closely related to the efficiency of photosynthesis and water use in plants [12–15]. Investigating the dynamics of plant architecture as quantified by these traits holds significant importance and can provide new insights for breeding superior varieties.

Tomato growth and development is a dynamic and complex process coregulated by genes and the environment. Its plant architectural traits are controlled by numerous genes/quantitative trait loci (QTLs), most of which have small or moderate effects. The genetic and temporal control mechanisms of these traits at specific stages are largely unknown [16, 17]. Additionally, tomato plant architecture is influenced by agricultural practices such as fertilization and environmental factors such as soil water content. Therefore, tomato breeding often requires the use of heritability analyses to predict trait genetic stability, helping reveal the genetic basis and understand the dynamic interaction of genes with the environment [18, 19]. While recent advances in tomato genome sequencing and high-throughput genotyping have greatly facilitated the genotyping of numerous lines to detect the genetic basis of plant architecture variation, large-scale, rapid, and accurate phenotyping in the field remains a major bottleneck in the genetic study of plant architecture dynamics [20–23]. Traditional phenotyping is laborious, costly, and inefficient, resulting in most tomato architecture studies focusing on a specific developmental stage and/or a single trait, despite the known changes in gene expression patterns during plant development. The resultant lack of data on temporal changes of plant architectural traits undoubtedly limits the use of temporal heritability analysis and therefore prevents deeper understanding of temporal variations in gene expression heterogeneity [3]. In contrast, high-throughput phenotyping platforms (HTPs) offer opportunities to capture a large number of plant architectural traits across multiple developmental stages, addressing the phenotyping bottleneck and revealing the temporal genetic control underlying crop growth and development. For example, in commercialized large-scale tomato greenhouses with densely planted rows and overlapping canopies, unmanned ground vehicles (UGVs) integrated with RGB-D cameras can capture high-frame rate, multiview RGB-D images and reconstruct 3D point clouds of tomato plants at different stages [24–26]. Compared to fixed sensor arrays, automated track systems, and unmanned aerial vehicles (UAVs), the UGV offers greater flexibility, enabling free movement within the greenhouse and comprehensive coverage of different plant growth areas, thereby overcoming the limitations imposed by spatial layout and crop configuration [27]. Point cloud-based methods allow for automatic, objective, precise, and continuous quantification of plant architectural traits [28–30]. This UGV-based proximal sensing solution offers new opportunities for accurate and efficient characterization of plant architecture dynamics as well as temporal heritability analysis.

Organ segmentation is a critical step in extracting plant architectural traits from the massive raw data obtained by UGV phenotyping platforms. Accurately decomposing complex plant structures into individual organs, such as stems, leaves, flowers, and fruits, is a prerequisite for the precise measurement of each organ’s geometric characteristics (e.g. size, shape, position), spatial relationships, and topological structures. Unlike object detection models, which are limited to determining organ classification and localization at the instance level, segmentation models enable pixel-level or voxel-level classification, resulting in more precise geometries and topological relationships [31, 32]. Compared to direct point cloud segmentation, image segmentation has undergone extensive research and optimization, allowing for higher accuracy at lower computational costs. Additionally, point clouds generated directly from depth images are often sparse, which further complicates point cloud segmentation. As a result, plant organs are typically segmented at the RGB image level first, and then point cloud reconstruction is performed using depth images. Prior to the widespread adoption of deep learning techniques, RGB image segmentation mainly relies on traditional methods such as edge detection, region growing, cluster analysis, and threshold segmentation, which are greatly affected by noise and lighting changes and difficult-to-deal-with complex greenhouse scenes [33, 34]. Deep learning, on the other hand, automatically learns image features through large-scale data training, with higher segmentation accuracy and stability. Currently, mainstream deep learning-based RGB image segmentation models primarily use the convolutional neural network (CNN) or transformer architectures as backbones, and their segmentation accuracies can be improved by an iterative process [35–39]. However, constrained by the single modality of RGB data, these models may suffer from semantic confusion in complex greenhouse environments [40]. To address this issue, multispectral (MS) and depth (D) images have been introduced into the image segmentation field [41]. MS images capture spectral information beyond the visible range, providing additional insights into crop physiological and biochemical characteristics. D images contain richer spatial information, enabling a better understanding of the 3D structure of plant organs. These complementary spectral and spatial features help overcome the limitations of single-modality RGB image segmentation [42–44]. In addition, using a single network to extract features from different modalities and fuse them at the input stage, or deploying two separate backbone networks to independently extract features from different modalities and then fuse them, are typically tailored for specific modality pairs (e.g. RGB-D or RGB-T) [45–49]. However, these approaches still face significant challenges when dealing with complex multimodal data, such as the fusion of RGB, MS, and D information. Therefore, it is necessary to design multimodal image fusion strategies, and segmentation models for MS and RGB-D data to achieve more accurate and robust organ segmentation in greenhouse tomatoes.

The key plant architectural traits of tomato encompass a variety of parameter types, such as distance, angle, area, volume, etc. Accurate extraction of these traits requires the deep integration of methods from multiple disciplines, such as geometry, topology, and statistics [50]. Directly transferring existing plant architectural trait extraction models presents significant limitations. Due to the unique structural characteristics of tomato plants, models developed for other crops (such as cotton, maize, sorghum, etc.) often cannot be directly applied [51–53]. Furthermore, existing tomato-specific models, if directly used in greenhouse environments, may suffer from issues such as the failure of morphological simplification assumptions, limited trait types, and applicability restricted to specific growth stages [54–57]. Therefore, it is essential to develop accurate and robust extraction methods tailored to both the biological characteristics of greenhouse tomatoes and the unique conditions of greenhouse environments, capable of handling a wide range of plant architectural traits across multiple growth stages.

This study aims to propose a novel UGV-based approach for high-throughput phenotyping of tomato architectural traits. The specific objectives of this study are to (a) design and implement a novel UGV plant phenotyping system for automated navigation and data collection, (b) develop a SegFormer-based model to fuse multispectral and depth images (MSD-SF) for plant organ segmentation, and (c) extract six key tomato architectural traits (i.e. ST, LS, IH, FS, TLA and LIA) from the reconstructed organ point cloud. Results indicate that the proposed phenotyping pipeline can accurately measure plant architectural traits and map the temporal dynamics of their broad-sense heritability. Overall, our method will provide affordable, flexible, comprehensive, and automated phenotyping measurements of tomato plant architectural traits and enable successive genetic studies of spatiotemporal changes of tomato plant architecture in controlled environments.

## Results and discussion

### High-throughput phenotyping pipeline of tomato architectural traits

We proposed a novel pipeline for high-throughput phenotyping of tomato architectural traits (Fig. S1). The key steps are the collection of multimodal image data including RGB-D and MS images of greenhouse tomato plants using a self-developed UGV plant phenotyping platform, the segmentation of the tomato organ instances with MSD-SF and point cloud clustering, and the estimation of six key traits (ST, LS, IH, FS, TLA, and LIA). We used time cost to evaluate the efficiency of the data collection, employed mean intersection over union (mIoU) and mean pixel accuracy (mPA) to assess the segmentation performance, and assessed the estimation accuracy of the traits using the coefficient of determination (R^2^), root-mean-square error (RMSE) and relative root-mean-square error (rRMSE). Finally, we extracted the plant architectural traits of different tomato varieties at different growth stages using the proposed method, and plotted the temporal variation of broad-sense heritability.

### Efficiency of data collection

Figure S2a presents an example of a 2D occupancy grid map of the greenhouse scene constructed by the UGV for tomatoes at the seedling stage. Pixels in deep blue color indicate the occupied points, white pixels denote the unoccupied points, and other pixels represent unknown areas. Figure S2b and c, respectively, illustrates the UGV navigation route and the actual waypoints during data collection. The waypoints on both sides of a single plant showed a centrosymmetric distribution, which reduced the overlap of the field of view (FOV) from different viewpoints and thus improved the overall coverage of the canopy. Field tests demonstrated that the UGV could complete all greenhouse imaging tasks within 2.5 h. The average data collection time per tomato plant was 30 s. In contrast, manual measurements took 3–5 min per tomato plant (with at least 3 repetitive sampling measurements for each trait), which is much slower compared to UGV measurements.

Waypoint-based multiview image acquisition tasks in complex greenhouse environments are challenging due to the need for robust navigation, efficient data collection, and practical deployment. Unlike wheeled or legged robots, our tracked UGV excels in maneuvering over uneven terrain and navigating narrow row spaces [59, 60]. It offers simple control, low cost, and is equipped with light detection and ranging (LiDAR) and obstacle avoidance radar for precise localization and mapping, as well as detecting road encroachments by extending branches [58, 61]. The UGV improves data acquisition efficiency by adjusting the camera pose only at the initial waypoint, eliminating the need for adjustments at each waypoint [62]. However, reliance on outdated 2D grid maps can limit navigation performance. Frequent rescanning to update the map is inefficient, so we recommend updating maps weekly during the vegetative and early flowering stages of tomatoes, and biweekly during other periods. Future work should focus on autonomous exploration and dynamic map updating to improve efficiency. The resolution of the LiDAR sensor is also critical, as low-resolution sensors may fail to detect small stems during the seedling stage. A minimum horizontal angular resolution of 0.1° and centimeter-level distance accuracy are essential for precise obstacle detection. Incorporating vision-assisted navigation could further enhance system robustness and adaptability [63, 64].

### Segmentation performance

**Figure 1:**
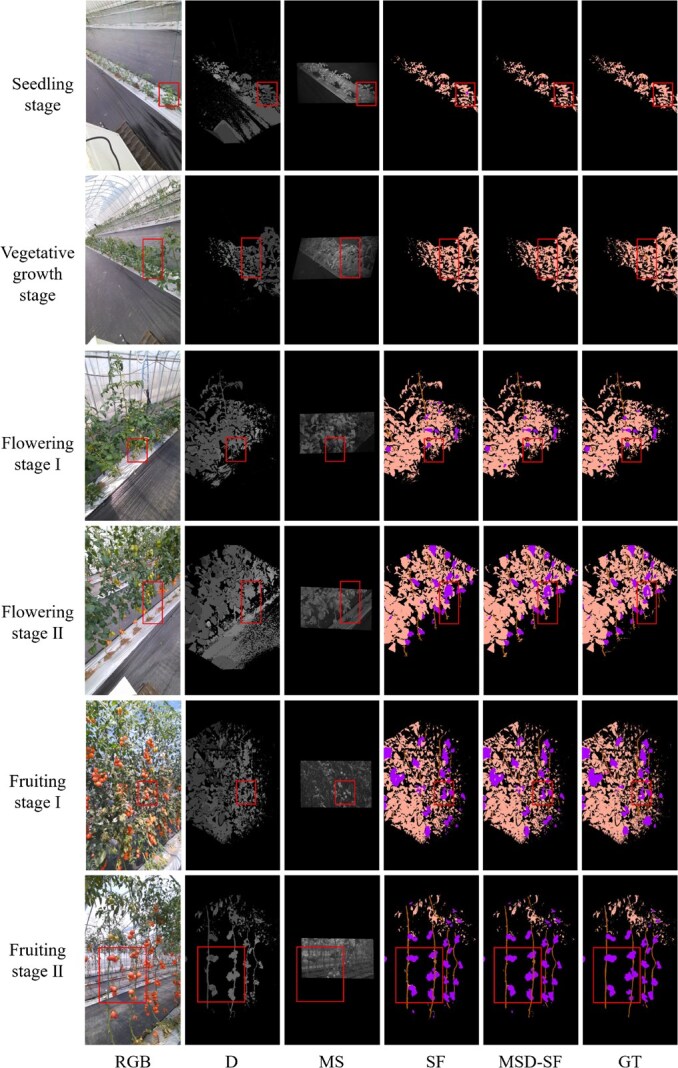
Multimodal images of tomato plants at different growth stages and their semantic segmentation results. Flowering stage I, early flowering stage; Flowering stage II, late flowering stage; Fruiting stage I, early fruiting stage; Fruiting stage II, late fruiting stage; D, depth images; MS, multispectral images (740.7 nm); SF, segmentation results of SegFormer; MSD-SF, segmentation results of the SegFormer with fusion of multispectral and depth modalities; GT, manually segmented ground truth.

Multimodal images of tomato plants at different growth stages and their semantic segmentation results are shown in Fig. 1. As only RGB images were used for training, the segmentation results of SegFormer showed some misclassifications. It was observed that SegFormer identified fruits that were not present in the seedling stage, failed to recognize stems within the canopy, and exhibited imprecise boundaries even though the categories were roughly correct. In contrast, MSD-SF trained with multimodal images resulted in less misclassification and more precise boundaries in the segmentation results.

The quantitative results of average segmentation performance for MSD-SF and other models are presented in Table 1 (see more detailed results of the 4-fold cross-validation in Table S1). The addition of input data modalities significantly improved the semantic segmentation performance. Compared to SegFormer using RGB images as input and cross-modal fusion for RGB-X (CMX) [65] using RGB-D images as input, MSD-SF with RGB-D and MS image inputs showed an improvement of 8.00% and 3.05% in mIoU, respectively, and 6.27% and 1.95% in mPA, respectively. Among all segmentation results, the segmentation accuracy of leaves and fruits was higher, followed by stems, with flowers having the lowest accuracy. Specifically, MSD-SF achieved a nearly 2-fold increase of IoU in flower segmentation compared to SegFormer, leading to the most significant improvement in the segmentation accuracy among all organ types.

**Table 1:** Comparison of average segmentation performance of MSD-SF with other models

Model	IoU (%)				mIoU (%)	mPA (%)
	Stem	Fruit	Leaf	Flower		
SF	54.27	76.61	84.98	24.00	67.23	75.11
CMX	59.41	82.09	85.92	36.20	72.18	79.43
MSD-SF	**62.37**	**85.46**	**87.32**	**42.06**	**75.23**	**81.38**

SF, SegFormer; CMX, cross-modal fusion for RGB-X; MSD-SF, SegFormer with fusion of multispectral and depth modalities; IoU, intersection over union; mIoU, mean intersection over union; mPA, mean pixel accuracy.

To demonstrate the indispensable role of each component within the multiband fusion module (MFM) in enhancing semantic segmentation performance, we conducted an ablation study. We evaluated the mIoU with different inputs and modules of MFM, and the results of the ablation study are reported in Table 2. When using the pseudo-multispectral images with only the 666.5 nm band as input, the resulting mIoU was only 0.69% higher than that of the CMX segmentation results. This suggests that spectral information is crucial for enhancing segmentation performance, and simply adding a viewpoint with minimal difference offered limited improvement. The continuous convolution layers within the spatial-wise rectification module contributed to establishing receptive fields, facilitating the correlation of neighboring pixels. This mechanism assisted in the fusion of features from partially misaligned regions [66, 67]. It indicates that regardless of the fusion method used, removing spatial-wise rectification resulted in a slight decrease in segmentation performance, with a more pronounced decline in the segmentation of stems and flowers compared to fruits and leaves. This suggests that spatial-wise rectification is particularly beneficial for small-sized multimodal targets. Furthermore, the network parameters within the channel-wise fusion module, being updated automatically through learning, aided in exploring optimal nonlinear channel fusion methods. In contrast, linear fusion methods such as principal component analysis (PCA) are limited to simple linear combinations of channels, failing to model the nonlinear relationships between them. This limitation significantly diminishes their ability to extract essential feature information. Experimental results demonstrate that in segmentation tasks for stems, fruits, and flowers, the performance of PCA-based fusion is substantially inferior to that of nonlinear channel-wise fusion. This further underscores the superiority of nonlinear fusion strategies in feature extraction and their critical role in achieving robust segmentation outcomes. The performance of leaf segmentation does not show a significant overall decrease, possibly because leaves, being the dominant structure of the canopy, have large-scale spatial continuity and occupy a higher pixel proportion, which reduces the sensitivity of the segmentation task to the channel fusion method. On the other hand, the segmentation of smaller scale targets such as stems and flowers relies more on the cross-channel nonlinear interactions to enhance local detail features.

**Table 2:** Ablation study of MFM

Modules			Inputs		IoU (%)				mIoU (%)
Spatial-wise rectification	Channel-wise fusion	PCA-based fusion	RGB + MS	RGB + PMS	Stem	Fruit	Leaf	Flower	
√	√		√		**62.37**	**85.46**	**87.32**	**42.06**	**75.12**
√	√			√	59.39	82.65	86.54	36.81	72.87
√		√	√		56.59	78.35	85.68	27.87	68.98
		√	√		53.14	76.92	85.04	23.46	66.73
	√		√		60.03	84.72	86.46	38.28	74.31

MFM, multi-band fusion module; PCA-based fusion, principal-component-analysis-based fusion (see Method S1 for fusing multichannel images by extracting the first three principal components); RGB + MS, red, green, blue and multispectral images (650–950 nm, 25 bands); RGB + PMS, red, green, blue and pseudo-multispectral images (only 666.5 nm, 25 redundant bands); IoU, intersection over union; mIoU, mean intersection over union.

**Figure 2:**
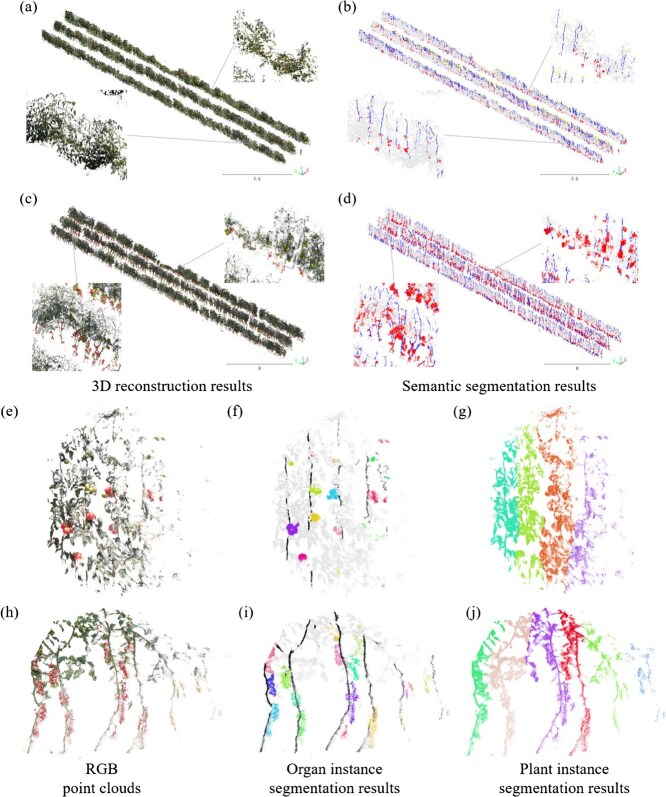
Tomato point clouds and segmentation results (see Figs S3 and S4 for all time period results). (a, c) RGB point clouds of the whole scene. (b, d) Semantic point clouds of the whole scene. (e, h) Localized RGB point clouds. (f, i) Point cloud instances of flower or fruit clusters. (g, j) Point cloud instances of tomato plants.

Figure 2 shows RGB point clouds, semantic point clouds, and point cloud instances of tomato organs (segmentation results for all periods are in Figs S3 and S4). Leaf segmentation was the most accurate due to the dense canopy, followed by fruit segmentation, which was aided by the distinct shape and color of fruits. Stems and flowers, sharing similar colors with leaves (especially in sunlight) and often shaded, had lower segmentation precision. The depth information in MSD-SF effectively addressed spatial occlusion and color similarities by providing relative positional and geometric features. For example, the spherical shape of fruits is distinguished by local curvature in the depth channel, and the cylindrical stem structure is captured through vertical continuity. Multispectral information added to MSD-SF overcame visible light limitations, enhancing the segmentation of small flowers. The multimodal data fusion module strengthened feature representation by combining spatial constraints from depth and material differences from spectral information. This enabled more accurate segmentation, such as during fruit enlargement, where spectral changes and depth information supported robust results. Distance-based clustering effectively segmented flowers, fruits, and stems. Flowers and fruits usually grow in clusters and stems are highly concentrated in their projections on the vertical plane, despite the discontinuities due to leaf shading. Overall, the proposed semantic segmentation model and clustering algorithm performed well on the tomato dataset obtained at different growth stages.

Our results demonstrated the effectiveness of using multimodal image fusion to address the challenge of low segmentation accuracy for crop organs in complex greenhouse environments. While the integration of an MS camera inevitably increased system costs, the enhanced segmentation accuracy proved highly beneficial for the 3D reconstruction of crop organs and the precise extraction of plant architectural traits. Additionally, by fusing D images, MS images, and semantic information from MSD-SF outputs, novel 3D multispectral point cloud data of plant organs can be easily generated, with each point containing both spatial coordinates and spectral reflectance. This enables the simultaneous extraction and analysis of 3D structural phenotypic traits and the physiological and biochemical functions of plant organs, offering a powerful tool for research in crop biology, genetics, and breeding. This represents another highly promising future application for our method.

### Estimation accuracies of tomato architectural traits

**Figure 3:**
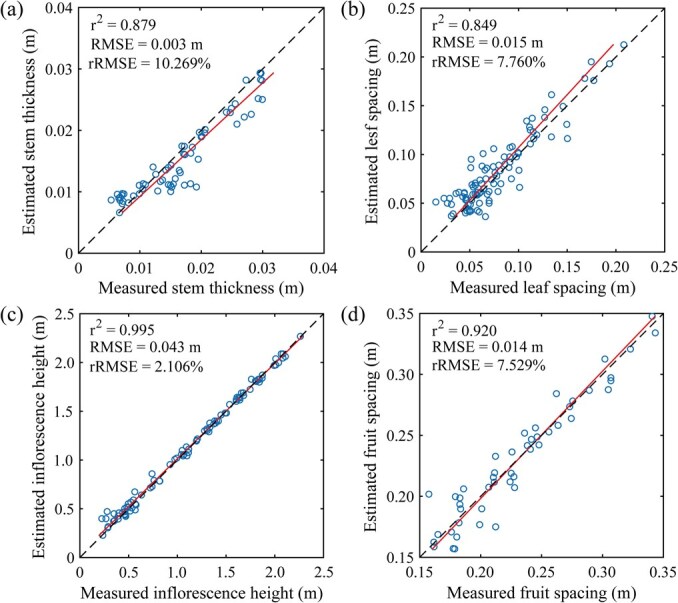
Accuracy of estimation for four types of tomato architectural traits. (a) Stem thickness. (b) Leaf spacing. (c) Inflorescence height. (d) Fruit spacing.

Figure 3 presents the estimation accuracy of ST, LS, IH, and FS. Overall, the proposed algorithms performed well in estimating four tomato architectural traits with R^2^ values all exceeding 0.8 and rRMSE values all <10%. Regarding goodness of fit, IH exhibited the highest level, with an R^2^ value close to 1, followed by FS and ST, while LS had the lowest R^2^ value, ~0.85. In terms of precision, IH also demonstrated the highest estimation precision, with an rRMSE of only ~2%. Slightly lower precision was observed for FS and LS. ST exhibited the lowest estimation precision, with an rRMSE of ~10%.

The accuracy of trait estimation is influenced by both the precision of organ segmentation and the performance of the trait extraction algorithm. The estimated values of ST were generally smaller than the ground truth (GT) (Fig. 3a). This discrepancy primarily arose because the predicted stem mask region might not extend beyond the GT, resulting in the loss of some edge pixels. Conversely, when the predicted mask region exceeded the GT, excess pixels were filtered out using a depth threshold, leading to a smaller but more accurate estimation that overlapped with the GT. This difference in mask regions also contributed to errors in the FS estimation (Fig. 3d). Regardless of whether the predicted mask region exceeded the GT, it could affect the accuracy of fruit cluster centroid estimation. This error was particularly evident in cherry tomatoes with larger fruit clusters. Additionally, occlusion from dense canopy layers could also affect the accuracy of fruit cluster centroid estimation. The estimated values of LS were generally larger than the GT (Fig. 3b). This might be due to missing leaves, resulting in an overestimation of LS. The occlusion of stem nodes by dense canopy layers made it challenging to directly detect stem nodes [36, 68]. Thus, using LS, which was highly correlated with the internode length (IL), as an indirect predictor, was a feasible alternative. However, this still needed to consider the errors introduced by the diverse morphology of leaves. The estimation of IH was relatively accurate (Fig. 3c), but the low accuracy of inflorescence segmentation might still affect its practical application. The estimated values of TLA were usually underestimated due to the influence of canopy occlusion. LIA estimation was closely related to the estimation of leaf normal vectors. However, the latter was highly sensitive to point cloud density and often performed poorly, especially at leaf edges where data might be sparse.

Compared to existing sparse-view imaging methods, the approach proposed in this study not only ensures the accuracy of tomato plant architectural trait extraction but also significantly enhances the quantity and diversity of extractable traits. Yao et al. reconstructed point clouds from front and back view RGB-D images and extracted distance-related traits such as plant height and stem diameter through point cloud segmentation [69]. However, the types of traits they could extract were limited, and the manual camera movement coupled with point cloud segmentation resulted in high labor and computational costs. Cho et al. employed the YOLOR model for tomato organ detection, which only allowed the extraction of distance-related parameters such as stem diameter and the distance between branching points and growth points [62]. In contrast, our method integrates semantic segmentation and instance segmentation techniques, enabling the extraction of angle-related and area-related parameters, thereby significantly improving the comprehensiveness and diversity of trait extraction. Although our method exhibits slight limitations in the number, type, and accuracy of traits compared to dense multiview imaging methods, the practical feasibility of dense multiview imaging for large-scale greenhouse applications is constrained [29, 54–56, 70]. Our approach achieves an optimal balance between operational efficiency and trait estimation accuracy, demonstrating broader application potential.

### Temporal changes of tomato architectural traits

#### Temporal variation of ST, LS, IH, and FS during the entire growth period

**Figure 4:**
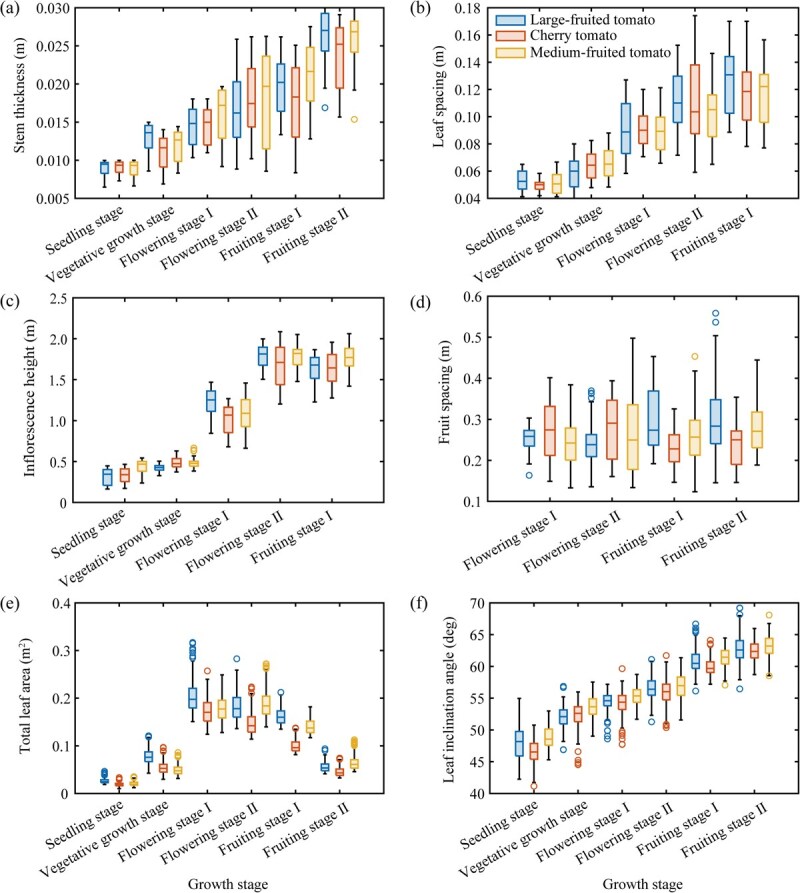
Boxplots of the tomato architectural traits for different fruit shapes at various growth stages (the line plots are presented in Fig. S5). (a) Stem thickness. (b) Leaf spacing. (c) Inflorescence height. (d) Fruit spacing. (e) Total leaf area. (f) Leaf inclination angle. The position and length of the boxes respectively indicate the overall mean and interspecies differences regarding specific traits. Flowering stage I, early flowering stage; Flowering stage II, late flowering stage; Fruiting stage I, early fruiting stage; Fruiting stage II, late fruiting stage.

ST was measured at all growth stages of tomatoes, as presented in Fig. 4a. Generally, ST demonstrated a gradually increasing trend, with interspecies differences initially increasing and then slightly decreasing. During the seedling stage, there was minimal variation in ST among tomatoes of different fruit shapes. During the subsequent growth stages, cherry tomatoes consistently exhibited smaller ST values compared to medium-fruited tomatoes. Large-fruited tomatoes showed higher ST values during the vegetative growth stage compared to other fruit shapes. However, their ST growth rate slowed down afterward, lagging behind other fruit shapes until regaining the lead during the fruiting stage.

LS was measured at all growth stages except for the late fruiting stage, as shown in Fig. 4b. Generally, LS demonstrated a gradual increase, with interspecies differences increasing until the flowering stage and then stabilizing. During the seedling stage, large-fruited tomatoes exhibited slightly larger LS values compared to other fruit shapes. However, during the subsequent stages, their LS values slightly decreased and then regained the lead after the late flowering stage. The LS values of cherry tomatoes and medium-fruited tomatoes were very close during all stages until slightly surpassing cherry tomatoes after the late flowering stage.

IH was measured at all stages except for the late fruiting stage, as presented in Fig. 4c. Overall, IH showed a significant increase during early and late flowering stages, stabilizing afterward. Interspecies differences enlarged at the beginning of the flowering stage and remained stable thereafter. Except for the early flowering stage, medium-fruited tomatoes consistently led in IH values compared to other fruit shapes during all stages.

FS was measured from early flowering to late fruiting stages, as shown in Fig. 4d. Generally, FS values ranged between 0.2 and 0.3 m, but with significant interspecies differences and variations. During the fruiting stage, large-fruited tomatoes demonstrated larger FS values and greater interspecies differences compared to the flowering stage. Medium-fruited tomatoes showed a slight increase in FS values in each period, with interspecies differences only enlarging during the late flowering stage, and remaining stable in other stages. The FS values and interspecies differences of cherry tomatoes during the fruiting stage were smaller compared to the flowering stage.

#### Visualization of TLA and LIA during the entire growth period

TLA was measured at the entire growth stage, as presented in Fig. 4e. Generally, TLA values and interspecies differences showed an increasing trend followed by a decrease, peaking at the flowering stage. Except for the late flowering and late fruiting stages, large-fruited tomatoes consistently exhibited the highest TLA values, surpassing medium-fruited tomatoes in all other stages. Cherry tomatoes showed the smallest TLA values, except during the vegetative growth stage, where they surpassed medium-fruited tomatoes. LIA was measured at all growth stages, as shown in Fig. 4f. Generally, LIA values showed a gradual increase, with stable interspecies differences. Medium-fruited tomatoes consistently exhibited the highest LIA values in all stages, while cherry tomatoes showed the lowest values except during the vegetative growth stage, where they surpassed large-fruited tomatoes. The qualitative results of TLA and LIA are illustrated in Fig. S6, showing trends in the number of points and N_z_ (z-axis component of the leaf normal vector, cosine value of LIA) consistent with the quantitative analysis results in Fig. 4e–f.

### Temporal changes of broad-sense heritability

**Figure 5:**
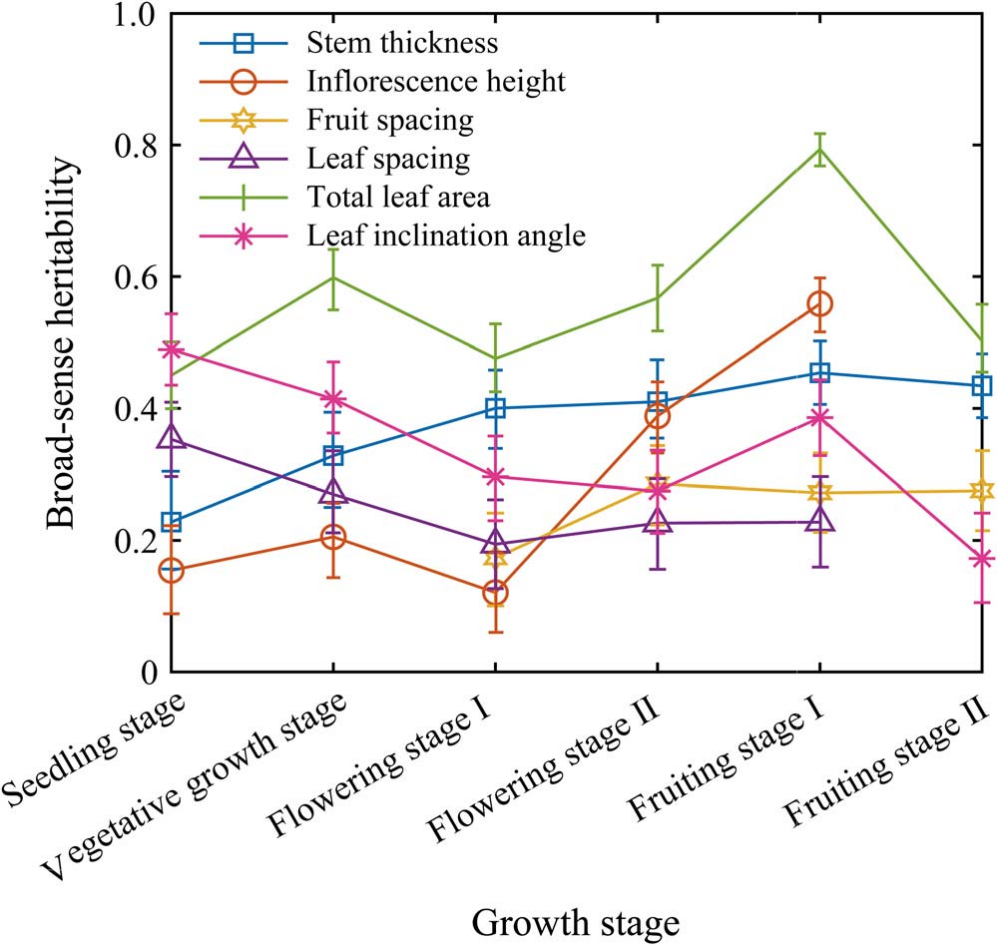
Temporal changes in broad-sense heritability of various architectural traits in tomato plants across growth stages. Flowering stage I, early flowering stage; Flowering stage II, late flowering stage; Fruiting stage I, early fruiting stage; Fruiting stage II, late fruiting stage.

Figure 5 presents the line graph illustrating the temporal changes in broad-sense heritability of various architectural traits in tomato plants across growth stages (see Table S2 for corresponding statistical test results). Broad-sense heritability of ST, IH, and FS generally showed an increasing trend during the trait’s existence period. The broad-sense heritability of ST showed an upward trend before the fruiting stage, stabilizing afterward within the range of 0.4–0.5. IH’s broad-sense heritability fluctuated at low levels >0.1–0.2 before the early flowering stage but significantly increased to 0.56 during the early fruiting stage. FS’s broad-sense heritability increased from 0.17 during the flowering stage to 0.29 and remained relatively stable thereafter. In contrast, LS and LIA exhibited a decreasing trend in broad-sense heritability during the trait’s existence period. LS’s broad-sense heritability decreased from 0.35 during the seedling stage to 0.19 during the early flowering stage, slightly increasing to 0.22 thereafter. LIA decreased from 0.49 during the seedling stage to 0.17 during the late fruiting stage, with a minor rebound to 0.39 only during the early fruiting stage. TLA’s broad-sense heritability fluctuated in an overall M-shaped pattern during the trait’s existence period. Its value increased from 0.45 during the seedling stage to 0.60 during the vegetative growth stage, then declined to 0.48 during the early flowering stage. The second peak occurred during the early fruiting stage at 0.79, followed by a decrease to 0.50 thereafter.

The temporal changes in broad-sense heritability of tomato architectural traits (shown in Fig. 5) are essentially driven by the sequential variations in phenological characteristics and environmental adaptability among different genotypes of tomatoes. This process involves a plethora of molecular components, physical factors, and complex regulatory interactions [71]. For example, plant architecture-regulating genes control the levels of various hormones, such as gibberellins and auxins, thereby regulating cell division and differentiation in different types of meristematic tissues. [2, 72, 73]. The temporal variation graph of broad-sense heritability generated in this study provides a more intuitive understanding of the selective expression of plant architecture-regulating genes throughout the entire typical growth cycle of tomato plants. When broad-sense heritability increased, the differences in the expression levels of architectural control genes and hormone levels among different genotypes of tomatoes gradually became significant, directly impacting the size, maturity, and activity of these meristematic tissues [17]. As the interspecific differences in the shoot apical meristem (SAM), axillary meristem (AM), and vascular cambium in promoting vertical growth, lateral growth, and inflorescence initiation of plants increased, the interspecific variance of traits such as ST, IH, FS, and LS during the flowering period also increased [74, 75]. Similarly, interspecific differences in plastochron and phyllochron also affected the rate of new leaf production, thereby increasing the interspecific variance of early TLA in tomatoes [76]. When broad-sense heritability decreased, influenced by the environment, the expression levels of architectural control genes and hormone levels in tomatoes tended to converge [77]. This induced changes in architectural traits toward environmental adaptation, resulting in reduced interspecific differences [78]. For example, competition among tomato plants for sunlight intensified with increasing canopy width [79]. To obtain optimal light interception and gain a competitive advantage over neighboring plants, they collectively chose to increase leaf inclination angles to acquire more lateral light [4, 5]. The weak light environment caused by canopy shading also promoted elongation of the main stem [80]. This convergence reduced the interspecific variance of early LS and overall LIA in tomatoes.

This study developed a novel and applicable approach for high-throughput extraction of tomato plant architecture traits as well as analysis of temporal broad-sense heritability, revealing the genetic contribution to these traits throughout the growth cycle. It provides breeders with a foundation for targeted selective breeding. The method can also be extended to different environments or stress conditions to identify genotypes sensitive to environmental factors, with the UGV system facilitating continuous trait monitoring. Additionally, as the tomatoes used in this study were all first-generation hybrids, future research could expand to include different generational lines. By quantitatively analyzing the correlation between the plant architecture traits of parents and offspring, the contribution of genetic factors to trait variation can be further assessed. This would also facilitate the estimation of narrow-sense heritability, which particularly depends on the regression relationship between parent and offspring traits.

### Environmental applicability analysis

Our successful deployment of the UGV plant phenotyping system in the tomato greenhouse demonstrated its potential for stable operation and efficient acquisition of multimodal image data to extract plant architectural traits. In addition to tomatoes, the system can also be used for other greenhouse crops with similar morphological characteristics, such as cucumbers, eggplants, etc. The proposed multimodal data fusion and segmentation methods support researchers in adjusting the data modality types (e.g. thermal infrared, fluorescence images, etc.) based on different crops and tasks. The developed trait extraction process is also applicable to plant 3D point clouds obtained from other sensors, such as LiDAR and scanners. For similar studies, it is recommended that the adaptability of crop layout to environmental factors be fully considered during design and deployment. The configuration of row and plant spacing influences UGV maneuverability, with wide spacing facilitating movement and reducing collision risks but potentially lowering yield. Narrow row spacing, while increasing planting density and yield, demands compact UGV designs, increased energy consumption, and more frequent adjustments to UGV pose, raising collision risks. Therefore, full consideration should be given to the organic integration of agromechanics and agronomy. Lighting conditions, including strong natural light, can affect the accuracy of LiDAR and RGB-D cameras, leading to noisy or distorted data. To mitigate this, stable overcast weather or active lighting supplementation with dynamic radiometric calibration is recommended. For phenotyping tasks oriented toward environmental stresses, pests, and diseases that can change plant morphology, more datasets are needed to deal with changes in plant structure and fine-tuning methods are needed to address their uniqueness. These targeted adjustments help ensure the accuracy, efficiency, and scalability of the UGV plant phenotyping system when operating in diverse agricultural environments.

## Materials and methods

### Experimental design

The experiment was conducted in a plastic greenhouse located at Hangzhou Academy of Agricultural Sciences, Zhejiang Province, China. The layout of the greenhouse with a size of 40 m in length and 8 m in width is shown in Fig. S7a. Three planting rows were arranged in the greenhouse. Inter-row pathways and edges at both ends were provided for the U-turn of UGV. Thirty-three tomato varieties were used in this study as shown in Fig. S7b. Each row was planted with 11 tomato varieties with similar fruit size, with 10 plants of each variety. Large-fruited, cherry and medium-fruited tomatoes were planted in the first, second, and third rows, respectively. Except for the ‘903F1’ variety (the fifth variety in the first row), other varieties were indeterminate growth types. Tomato plants were sown and seedlings started in early January 2023, and were transplanted into the plastic greenhouse in early March and hung on vines. Pollination was conducted in early April, followed by regular pruning until early July when fruit harvesting was completed. Regular water and fertilizer management was applied during the whole experimental period.

### Data collection

#### UGV plant phenotyping system

Figure S8 illustrates the schematic diagram of image acquisition throughout the entire growth period using the self-developed UGV plant phenotyping system. The UGV hardware system consisted of a tracked chassis, LiDAR (RS-LiDAR-16, RoboSense, Shenzhen, China), obstacle avoidance radar (Mid-70, LIVOX, Shenzhen, China), navigation control module, robotic arm (UR5, Universal Robots, Odense, Denmark), RGB-D camera (Azure Kinect DK, Microsoft, Redmond, WA, USA), MS camera (MQ022MG-CM, XIMEA, Munster, Germany), 3D laser scanner (PRINCE775, SCANTECH, Hangzhou, China), and laptop computer [81]. The tracked chassis ensured the UGV’s maneuverability through the complex terrain inside the greenhouse. The navigation control module located at the front of the UGV was used for autonomous navigation and obstacle avoidance with real-time data analysis from the LiDAR and obstacle avoidance radar. A 6-degree-of-freedom robotic arm attached with imaging sensors through a custom-made connector was installed on the top platform of the UGV, which facilitates the transformation of camera poses to achieve multiview image acquisition. The laptop computer served as the host machine for the UGV plant phenotyping system, coordinating the overall control of its components. The navigation control module, robotic arm control box, and imaging sensors communicated with the host machine via Wi-Fi, Ethernet, and USB 3.0, respectively. A web-based software was also developed for the control of the entire system.

#### Route planning and image acquisition

We employed the preplanned waypoints and routes presented in Fig. S8a for automated data collection. The UGV started from the starting waypoint (origin) located near the main entrance of the greenhouse, navigated along a zigzag route to each predefined waypoint for static image collection, and then returned. For each line of the same tomato variety, two adjacent waypoints were set for UGV data collection. An extra waypoint was set at each end of the row to capture images of the hemispherical reference required for radiometric calibration [81]. As for navigation algorithms, we employed GMapping for UGV simultaneous localization and mapping (SLAM) and probabilistic roadmap (PRM) for path planning between adjacent waypoints [82, 83].

Multitemporal tomato image data were collected at six different growth stages, including seedling, vegetative growth, early flowering (flowering stage I), late flowering (flowering stage II), early fruiting (fruiting stage I), and late fruiting stages (fruiting II) (Fig. S8b). The image data included RGB-D images with a spatial resolution of 720 × 1280 pixels and MS images with 25 bands at 650–950 nm and a spatial resolution of 216 × 409 pixels. Before each experiment, the robotic arm’s pose was reset based on the canopy width and plant height, ensuring that all camera viewpoints covered the plant canopy as much as possible. Thereafter, the robotic arm maintained a fixed pose until it reached the starting waypoint, where a positional change was performed to acquire multiview images. The above method ensures that each tomato plant was covered by eight viewpoints. The D images extracted from the RGB-D images could be used to generate 3D point clouds of tomato plants. The remaining RGB and MS images together provided spectral information in the visible-near-infrared bands that could be used for multimodal image segmentation.

#### Ground truth data acquisition

We manually sampled and collected data on measurable tomato architectural traits from selected tomato plants as GT values for verifying the accuracy of architectural trait extraction. We measured IH, FS, and LS with a tape measure and measured ST with a Vernier caliper. Measurements were repeated five times per plant and averaged. For convenience, we used the spacing between the ends of neighboring petioles on the main stem as the ground true value of a single measurement of LS.

### Plant organ segmentation

#### Semantic segmentation using MSD-SF

**Figure 6:**
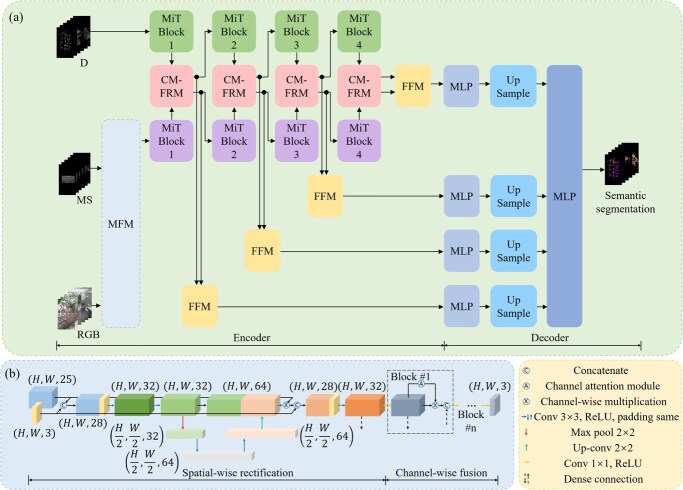
Schematic diagram of the SegFormer with fusion of multispectral and depth modalities (MSD-SF). (a) MSD-SF. (b) MFM. MS, multispectral images; D, depth images; MFM, multiband fusion module; MiT, Mix Transformer; CM-FRM, cross-modal feature rectification module; FFM, feature fusion module; MLP, multilayer perceptron; H, height; W, width.

A multimodal image semantic segmentation network called MSD-SF was designed for segmenting tomato plant organs (i.e. stem, leaf, flower, and fruit) as shown in Fig. 6a. MSD-SF adopted an encoder–decoder architecture that utilized a dual-stream hierarchical network for the encoder to extract features from both MS and D modal data and an all-multilayer-perceptron (All-MLP) design for the decoder to fuse multimodal and multilevel features and to predict semantic segmentation masks. Specifically, the D images directly entered one branch network in the encoder after the input layer. The RGB images and MS images registered through speeded-up robust features and Demons (SURF-Demons, see Method S2 for details) entered another branch network in the encoder after passing through an MFM [41]. We used the Mix Transformer encoder (MiT) from SegFormer (pretrained on ImageNet) as the backbone of MSD-SF [84, 85]. This backbone consisted of four consecutive transformer blocks with the same architecture, which can extract multilevel features of the same modal images. We also incorporated the cross-modal feature rectification module (CM-FRM) and feature fusion module (FFM) from CMX. The former was used to rectify the channel-wise and spatial-wise features of different modalities, while the latter was used to exchange and fuse features of different modalities. The fused multilevel features each passed through an MLP to unify the channel dimension. Subsequently, these features were concatenated together after passing through an upsampling layer. Finally, a series of MLPs were used to fuse the concatenated features and predict the segmentation masks.

The architecture design of the MFM is illustrated in Fig. 6b. MFM consisted of two stages: spatial-wise rectification and channel-wise fusion. In the spatial-wise rectification stage, the input RGB images and MS images were first concatenated along the channel dimension. After passing through two consecutive 3 × 3 convolutions, ReLU activations, and padding same operations, the tensor underwent downsampling using 2 × 2 max-pooling. Subsequently, after two more consecutive 3 × 3 convolutions, the tensor was upsampled and convolved again before concatenating with the previously unsampled tensor along the channel dimension. After that, channel-wise multiplication was performed on the multispectral input and the feature map obtained by the concatenated tensor through 3 × 3 convolution, and then the RGB input was concatenated along the channel dimension. Finally, 3 × 3 convolution was used to obtain a 32-channel tensor. In the channel-wise fusion stage, a series of blocks with identical architectures were designed to sequentially reduce the 32-channel tensor to 24, 12, 6, and 3 channels. These blocks utilized channel attention mechanisms to adjust channel weights and employed 1 × 1 convolutions to reduce the channel dimension. Additionally, Dense connections were employed to enhance feature reuse and computational efficiency [86].

Spatial-wise rectification stage:


(1) \begin{equation*} {F}_1= ReLU\left({Conv}_{3\times 3}\left( ReLU\left({Conv}_{3\times 3}\left( Concat\left({F}_{RGB},{F}_{MS}\right)\right)\right)\right)\right) \end{equation*}

(2) \begin{equation*} {F}_2={Upconv}_{2\times 2}\left( ReLU\left({Conv}_{3\times 3}\left( ReLU\left({Conv}_{3\times 3}\left({Maxpool}_{2\times 2}\left({F}_1\right)\right)\right)\right)\right)\right) \end{equation*}

(3) \begin{align*} {F}_3&= ReLU\big({Conv}_{3\times 3}\big( Concat\big({F}_{MS}\circledast ReLU\big({Conv}_{3\times 3}\big( Concat\big({F}_1,{F}_2\big)\big)\big),\notag\\&\quad{F}_{RGB}\big)\big)\big) \end{align*}

Channel-wise fusion stage:


(4) \begin{equation*} {X}_n= ReLU\left({Conv}_{1\times 1}\left( Concat\left({F}_3,{X}_0,{X}_1,\dots, {X}_{n-1},{X}_{n-1}\circledast SE\left({X}_{n-1}\right)\right)\right)\right) \end{equation*}

(5) \begin{equation*} {F}_{out}={X}_3 \end{equation*}

where ${F}_{RGB}$, ${F}_{MS}$, ${F}_1$, ${F}_2$, ${F}_3$, and ${F}_{out}$ denote RGB feature, MS feature, intermediate features 1–3, and output feature. ${F}_{RGB}\in{\mathbb{R}}^{H\times W\times 3}$, ${F}_{MS}\in{\mathbb{R}}^{H\times W\times 25}$, ${F}_1,{F}_2,{F}_3\in{\mathbb{R}}^{H\times W\times 32}$, ${F}_{out}\in{\mathbb{R}}^{H\times W\times 3}$. ${X}_n$ denote the feature in the n^th^ block of the channel-wise fusion stage, $\circledast$ signifies channel-wise multiplication, and $SE\left(\cdotp \right)$ denotes the squeeze-and-excitation operation utilized for channel attention extraction [87].

#### 3D reconstruction of tomato organs

**Figure 7:**
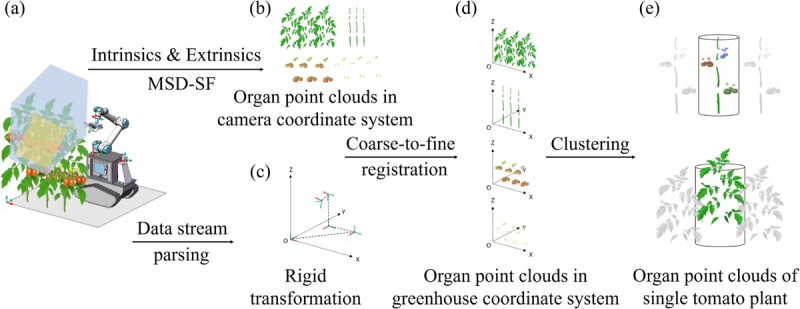
Schematic diagram of obtaining point cloud instances of tomato plant organs in the greenhouse coordinate system. (a) Coordinate systems of greenhouses and individual equipment and sensors. (b) Organ point clouds in the camera coordinate system. (c) Rigid transformation among different coordinate systems. (d) Organ point clouds in the greenhouse coordinate system. (e) Organ point clouds of the single tomato plant. MSD-SF, SegFormer with fusion of multispectral and depth modalities.

We developed a coarse-to-fine point cloud registration method (shown in Fig. 7) to obtain point cloud instances of tomato plant organs in the greenhouse coordinate system. Figure 7a illustrates the UGV capturing RGB-D images and MS images of tomato plants at a certain pose. The image data was segmented into masked images of tomato plant organs using MSD-SF. By combining the camera’s intrinsic parameters, the masked RGB-D images of organs were transformed from pixel coordinates to camera coordinates, resulting in organ point clouds (Fig. 7b). These point clouds were transformed from the camera coordinate system to the greenhouse coordinate system using a rigid transformation matrix between multiple coordinate systems (Fig. 7c). The specific mathematical derivation of the above two steps is detailed in Method S3. Subsequently, utilizing the Levenberg–Marquard iterative closest point (LM-ICP, see Method S4 for details) algorithm, the tomato plant organ point clouds in the greenhouse coordinate system were further finely registered (Fig. 7d) [88]. First, the point clouds from different views at the same waypoint were finely aligned (the target point cloud was the point cloud from the first view at that waypoint). After completing the point cloud alignment for each waypoint, the source and target point clouds were then set to be the point clouds of the current waypoint and the previous waypoint, respectively. The fine alignment started from the last waypoint and moved forward until it reached the first waypoint.

#### Organ point cloud instance segmentation

We utilized the Euclidean clustering algorithm to cluster individual organ instances from the point clouds of tomato plant organs and determine the plant affiliation of each organ instance (Fig. 7e). Specifically, we initially differentiated different stem instances based on the clustering results of stem point clouds projected onto the x–y plane. Subsequently, clustering was performed on the point clouds of flower and fruit organs to obtain their respective instances (instances obtained from fruit clustering represented individual fruit clusters). The plant affiliation of each organ instance was finally determined based on the distance between the centroids of organ instances and the centroid of stem instances along the vertical axis (for leaf instances, the determination was performed point by point).

### Architectural trait extraction

#### IH

**Figure 8:**
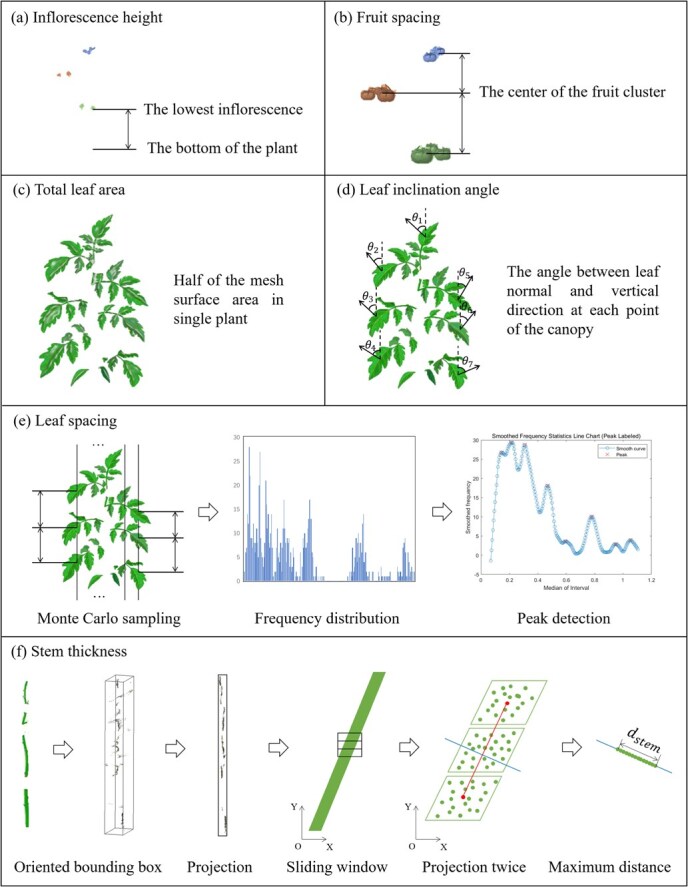
Schematic diagram of extracting tomato architectural traits. (a) Inflorescence height. (b) Fruit spacing. (c) Total leaf area. (d) Leaf inclination angle. (e) Leaf spacing. (f) Stem thickness.

IH was defined as the vertical distance between the lowest inflorescence and the base of the plant (Fig. 8a). This could be computed by calculating the difference between the minimum z-coordinate of the inflorescence centroid and the minimum z-coordinate of the plant point cloud.


(6) \begin{align*} IH&=\mathit{\min}\left\{\frac{1}{I_1}\sum_{i=1}^{I_1}{}_1{}^i{Z}_{inflorescence},\dots, \frac{1}{I_N}\sum_{i=1}^{I_N}{}_N{}^i{Z}_{inflorescence}\right\}\notag\\&\quad-\mathit{\min}\left\{{Z}_{plant}^1,\dots, {Z}_{plant}^M\right\} \end{align*}

where ${}_n{}^i{Z}_{inflorescence}$ represents the z-axis coordinate of the i^th^ point (${I}_n$ points in total) in the n^th^ inflorescence point cloud (*n* inflorescences in total) on the plant, and ${Z}_{plant}^m$ is the z-axis coordinate of the m^th^ point (M points in total) in the plant point cloud.

#### FS

FS was defined as the vertical spacing between adjacent fruit clusters (Fig. 8b). This could be obtained by calculating the difference between the z-axis coordinates of the centroids of adjacent fruit cluster point clouds.


(7) \begin{equation*} FS=\frac{1}{N-1}\sum_{n=1}^{N-1}\left(\frac{1}{I_{n+1}}\sum_{i=1}^{I_{n+1}}{}_{n+1}{}^i{Z}_{fruit}-\frac{1}{I_n}\sum_{i=1}^{I_n}{}_n{}^i{Z}_{fruit}\right) \end{equation*}

where ${}_n{}^i{Z}_{fruit}$ represents the z-axis coordinate of the i^th^ point (${I}_n$ points in total) in the n^th^ fruit cluster point cloud (*n* fruit clusters in total) on the plant.

#### TLA

The calculation method for TLA involved meshing the point cloud of leaves and then taking half of the total surface area of the mesh (Fig. 8c).


(8) \begin{equation*} TLA=\frac{1}{2}{S}_{leaf} \end{equation*}

where ${S}_{leaf}$ represents the total surface area of the meshed point cloud of leaves on the plant.

#### LIA

To obtain LIA, first, the normal vectors at each point of the leaf were estimated using the SVD method, and then redirected to the positive z-axis. Next, the angle between each point’s normal vector and the vertical direction was calculated (Fig. 8d).


(9) \begin{equation*} LIA=\frac{1}{M}\sum_{m=1}^M\left\langle{\boldsymbol{N}}^{\boldsymbol{m}},{\boldsymbol{Z}}_{+}\right\rangle =\frac{1}{M}\sum_{m=1}^M{\mathit{\cos}}^{-1}\left|{N}_z^m\right| \end{equation*}

where $\left|{N}_z^m\right|$ represents the z-coordinate of the redirected normal vector ${\boldsymbol{N}}^{\boldsymbol{m}}$ of the m^th^ point (M points in total) in the leaf point cloud.

#### LS

LS refers to the average vertical distance between adjacent leaves on the same stem. We proposed a novel method to calculate it, as illustrated in Fig. 8e. First, Monte Carlo sampling was applied to the leaf spacing, casting rays vertically within the canopy space and recording the distances between all ray and leaf intersections. Next, the frequencies of distances within each small interval were tabulated. A line graph was plotted with the interval midpoints and frequencies as the x and y coordinates, respectively, and the curve was smoothed. Then, a peak-finding algorithm based on derivatives was used to extract the x-coordinates corresponding to the peak values. Finally, the minimum value of half the x-coordinate and the difference between all adjacent x-coordinates were calculated, and the minimum of these values was taken as LS.


(10) \begin{equation*} LS=\mathit{\min}\left\{\frac{1}{2}{X}_{peak}^1,{X}_{peak}^2-{X}_{peak}^1,\dots, {X}_{peak}^{n+1}-{X}_{peak}^n,\dots, {X}_{peak}^N-{X}_{peak}^{N-1}\right\} \end{equation*}

where ${X}_{peak}^n$ represents the horizontal coordinate corresponding to the n^th^ peak value (*n* peaks in total).

#### ST

ST represents the average diameter of the stem, for which we devised a continuous projection method for computation (presented in Fig. 8f). Firstly, we utilized PCA to estimate the oriented bounding box (OBB) of the stem point cloud. Then, we selected the second-largest area face of the OBB for projection, establishing a Cartesian coordinate system with the long and short edges of that face as axes. Subsequently, we employed a 3 × 1 sliding window moving along the y-axis to extract three consecutive stem segments and computed the diameter of the middle segment during sliding. The specific approach involved calculating the axial direction of the middle stem segment based on the centroid connection line of the two side segments. Then, we projected the middle stem segment point cloud onto the normal direction of that axis. The maximum distance between points in the resulting 1D point cloud projection was taken as the diameter of the segment. Finally, the average of all segment diameters yielded the value of ST.


(11) \begin{align*} ST&=\frac{1}{M}\sum_{m=1}^M{d}_{stem}^m=\frac{1}{M}\sum_{m=1}^M\mathit{\max}\Big\{ dist\left({}_m{}^i{P}_{\mathit{\dim}1}-{}_m{}^j{P}_{\mathit{\dim}1}\right)|{i}_{max}\notag\\&={j}_{max}=N\Big\} \end{align*}

where ${d}_{stem}^m$ represents the diameter of the middle stem segment for the m^th^ sliding window (M windows in total). ${}_m{}^i{P}_{\mathit{\dim}1}$ and ${}_m{}^j{P}_{\mathit{\dim}1}$ denote the i^th^ and j^th^ points (*n* points in total) in the 1D point cloud computed for the m^th^ window.

**Table 3:** The distribution of the annotated tomato image dataset

Stages	Training set	Test set	Total
Seedling	32	8	40
Vegetative growth	32	8	40
Early flowering	24	6	30
Late flowering	24	6	30
Early fruiting	24	6	30
Late fruiting	24	6	30
Total	160	40	200

### Evaluation metrics

We evaluated the efficiency of the UGV-based data collection in terms of time cost. For organ segmentation, we annotated 200 images in total. The dataset distribution is shown in Table 3. Data augmentation methods including image rotation, image flipping, and brightness adjustment were used to expand the sample size. Four-fold cross-validation was also used to fully utilize the data. The MiT-B2 was employed as the pretrained weights of the backbone network. The embedding dimension of the MLP decoder was set to 512. Optimization was performed using the AdamW optimizer with a cross-entropy loss function, where the initial learning rate was set to 6.0 × 10^−5^ and a polynomial decay strategy (decay exponent of 0.9) was applied. Additionally, a linear learning rate warm-up over 10 epochs was implemented to ensure training stability. The training process spanned 200 epochs with a batch size of 2, incorporating weight decay (0.01) and batch normalization parameters (ε = 1.0 × 10^−3^, momentum = 0.1). The momentum coefficients for the optimizer and batch normalization layer were set to 0.9 and 0.1, respectively, ensuring stable gradient updates while maintaining flexibility in feature normalization. Using the hyperparameter settings described above, the MSD-SF model was trained on Google Colab for 10.5 h using one Tesla T4 GPU with 16.0 GB RAM and predicted masks at <1 s per frame. We employed mIoU and mPA to assess the segmentation performance of MSD-SF on the test set, and compared it with two baselines, SegFormer and CMX.


(12) \begin{equation*} mIoU=\frac{1}{k+1}\sum_{i=0}^k\frac{p_{ii}}{\sum_{j=0}^k{p}_{ij}+\sum_{j=0}^k{p}_{ji}-{p}_{ii}} \end{equation*}

(13) \begin{equation*} mPA=\frac{1}{k+1}\sum_{i=0}^k\frac{p_{ii}}{\sum_{j=0}^k{p}_{ij}} \end{equation*}

where k + 1 represents k target classes and 1 background class, ${p}_{ij}$ denotes the total number of pixels that belong to class i but are predicted as class j. Specifically, ${p}_{ii}$ represents true positives, ${p}_{ij}$ stands for false positives, and ${p}_{ji}$ represents false negatives. In terms of tomato architectural trait extraction, we deployed a Point Cloud Library-based C++ program using VS2019 on a computer with 16 GB of RAM and an Intel(R) Core (TM) i7–4790 CPU @ 3.60 GHz. The average elapsed time of the trait extraction algorithm was 81.3 s per plant. The estimation accuracy of some manually measurable traits was evaluated using R^2^, RMSE, and rRMSE. Due to variations in the existence time of different traits, we randomly collected 20 GT at each stage of trait existence and aggregated them into a test set.

We utilized the proposed methodology to extract the plant architectural traits of different tomato varieties at various growth stages, and generated a temporal change plot of broad-sense heritability ${H}^2$ for each trait.


(14) \begin{equation*} {H}^2=\frac{V_G}{V_P}\times 100\%=\frac{V_P-{V}_E}{V_P}\times 100\% \end{equation*}

where ${V}_P$ represents phenotypic variance, ${V}_G$ represents genetic variance, and ${V}_E$ represents environmental variance. Assuming that the estimate of a particular architectural traits for the n^th^ tomato plant (out of a total of *n* plants) of the m^th^ genotype (out of a total of M genotypes) at a particular stage is ${R}_m^n$. Then the ${V}_E$ and ${V}_P$ of this architectural trait can be calculated as follows:


(15) \begin{equation*} {V}_E=\frac{1}{M}\sum_{m=1}^M\left[\frac{1}{N-1}\sum_{n=1}^N{\left({R}_m^n-\frac{1}{N}\sum_{n=1}^N{R}_m^n\right)}^2\right] \end{equation*}

(16) \begin{equation*} {V}_P=\frac{1}{MN-1}\sum_{m=1}^M\sum_{n=1}^N{\left({R}_m^n-\frac{1}{MN}\sum_{m=1}^M\sum_{n=1}^N{R}_m^n\right)}^2 \end{equation*}

where M = 33 and *n* = 10 in this study. In addition, we estimated 95% confidence intervals (CIs) for broad-sense heritability and performed likelihood ratio tests (LRTs). The 95% CIs were estimated by bootstrap method by repeating 1000 samples from the original data in a relaxed manner, generating a simulated dataset, and then recalculating the target statistic (broad-sense heritability). The LRT is a test of whether the genetic variance is significantly nonzero by comparing the difference in likelihood values between the full model (with genotypic random effects) and the simplified model (without genotypic effects). The LRT statistic can be calculated using the following equation:


(17) \begin{equation*} LRT\ statistic=-2\times \left(\ln{L}_{Reduced}-\ln{L}_{Full}\right) \end{equation*}

where $\ln{L}_{Reduced}$ is the log-likelihood of the simplified model and $\ln{L}_{Full}$ is the log-likelihood of the full model. The *P*-value was then calculated from a chi-square distribution with one degree of freedom (the difference between the number of parameters of the full model and the number of parameters of the simplified model).

## Supplementary Material

Web_Material_uhaf109

## Data Availability

The pipeline code and example data related to this project are available as open source on GitHub (https://github.com/DigBigPigForU/Tomato-architectural-trait-extraction).
