# Supplementary material for: Phenotypic dynamics and temporal heritability of tomato architectural traits using an unmanned ground vehicle-based plant phenotyping system
Source: Hortic Res. 2025 Apr 30;12(8):uhaf109. doi: 10.1093/hr/uhaf109 (PMC12247514; doi:10.1093/hr/uhaf109)
Supplement: Web_Material_uhaf109 [file web_material_uhaf109.zip › S6.pdf]

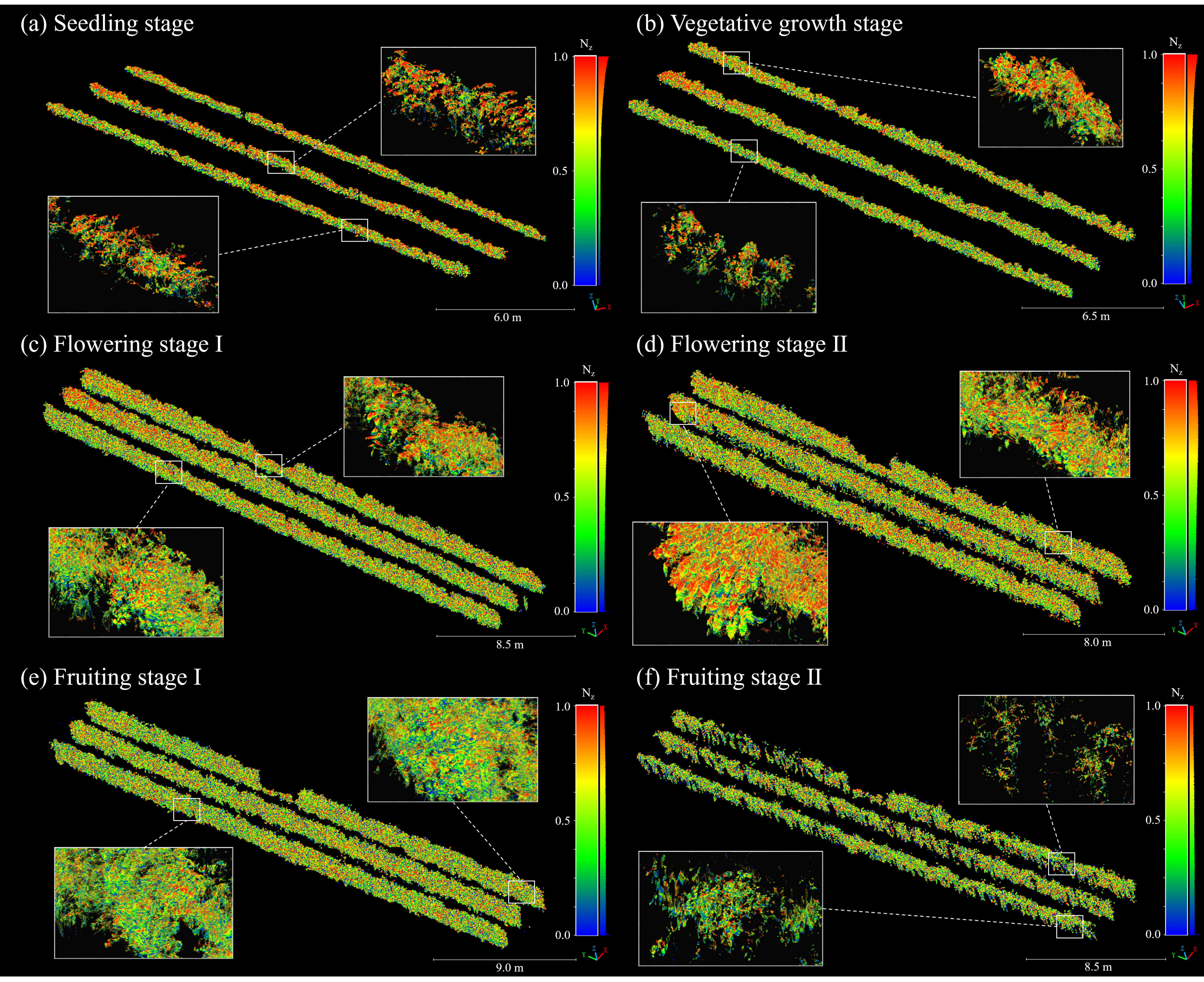
(a) Seedling stage
6.0 m
(b) Vegetative growth stage
6.5 m
(c) Flowering stage I
8.5 m
(d) Flowering stage II
8.0 m
(e) Fruiting stage I
9.0 m
(f) Fruiting stage II
8.5 m
N_z
1.0
0.5
0.0
