# Supplementary material for: Phenotypic dynamics and temporal heritability of tomato architectural traits using an unmanned ground vehicle-based plant phenotyping system
Source: Hortic Res. 2025 Apr 30;12(8):uhaf109. doi: 10.1093/hr/uhaf109 (PMC12247514; doi:10.1093/hr/uhaf109)
Supplement: Web_Material_uhaf109 [file web_material_uhaf109.zip › S7.pdf]

(a)

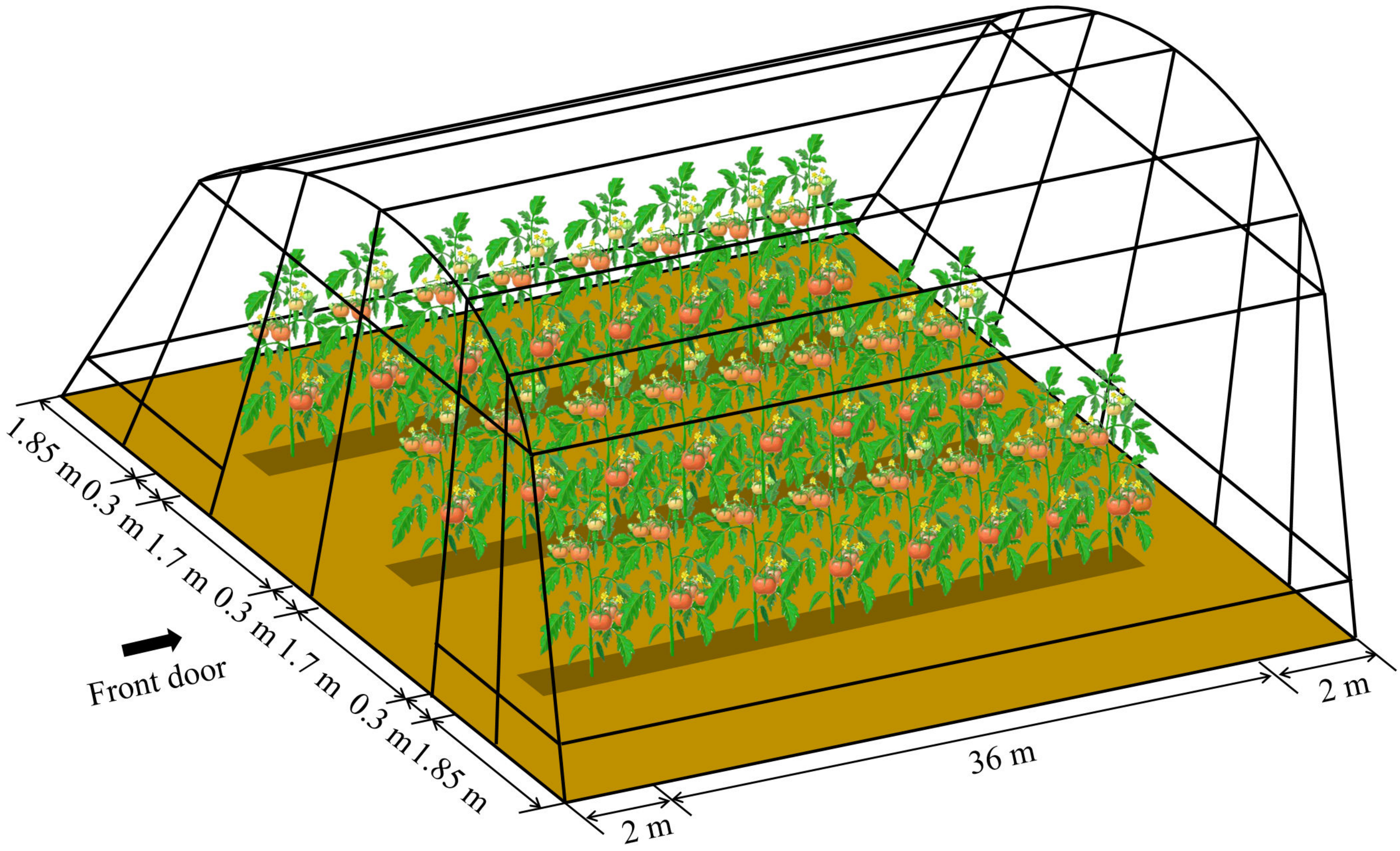

(b)

	Row 1	Row 2	Row 3
11	TY01F1	2117F1	Fencai No.5
10	T22007F1	Hangza503	Chengsemima
9	W05	18075*T11gd-116F8	Hangza701
8	W04	2228F1	583F1
7	TDL1618F1	2111F1	Zhefen202
6	R01	2114F1	Provence
5	903F1	T1745F1	Jiahong100
4	Zheza203	Huangfei	Hangza106
3	95102F1	Hangza504	Hangza603
2	Zidali	Hongfengling	Guanghui101
1	Hangza103	Hangza515	Hangza602
Front door			
