# Supplementary figures and images for: Phenotypic dynamics and temporal heritability of tomato architectural traits using an unmanned ground vehicle-based plant phenotyping system
Source: Hortic Res. 2025 Apr 30;12(8):uhaf109. doi: 10.1093/hr/uhaf109 (PMC12247514; doi:10.1093/hr/uhaf109)

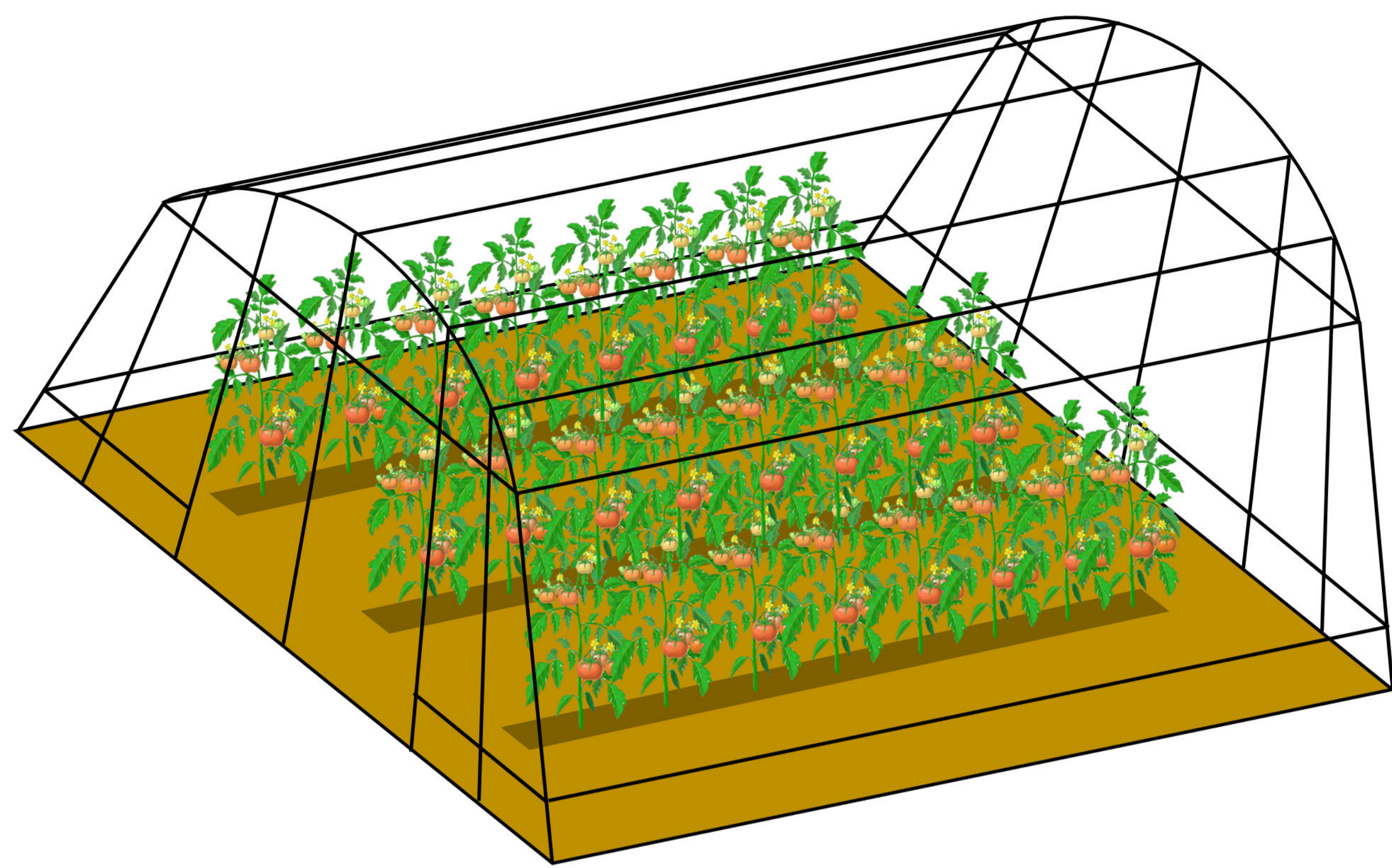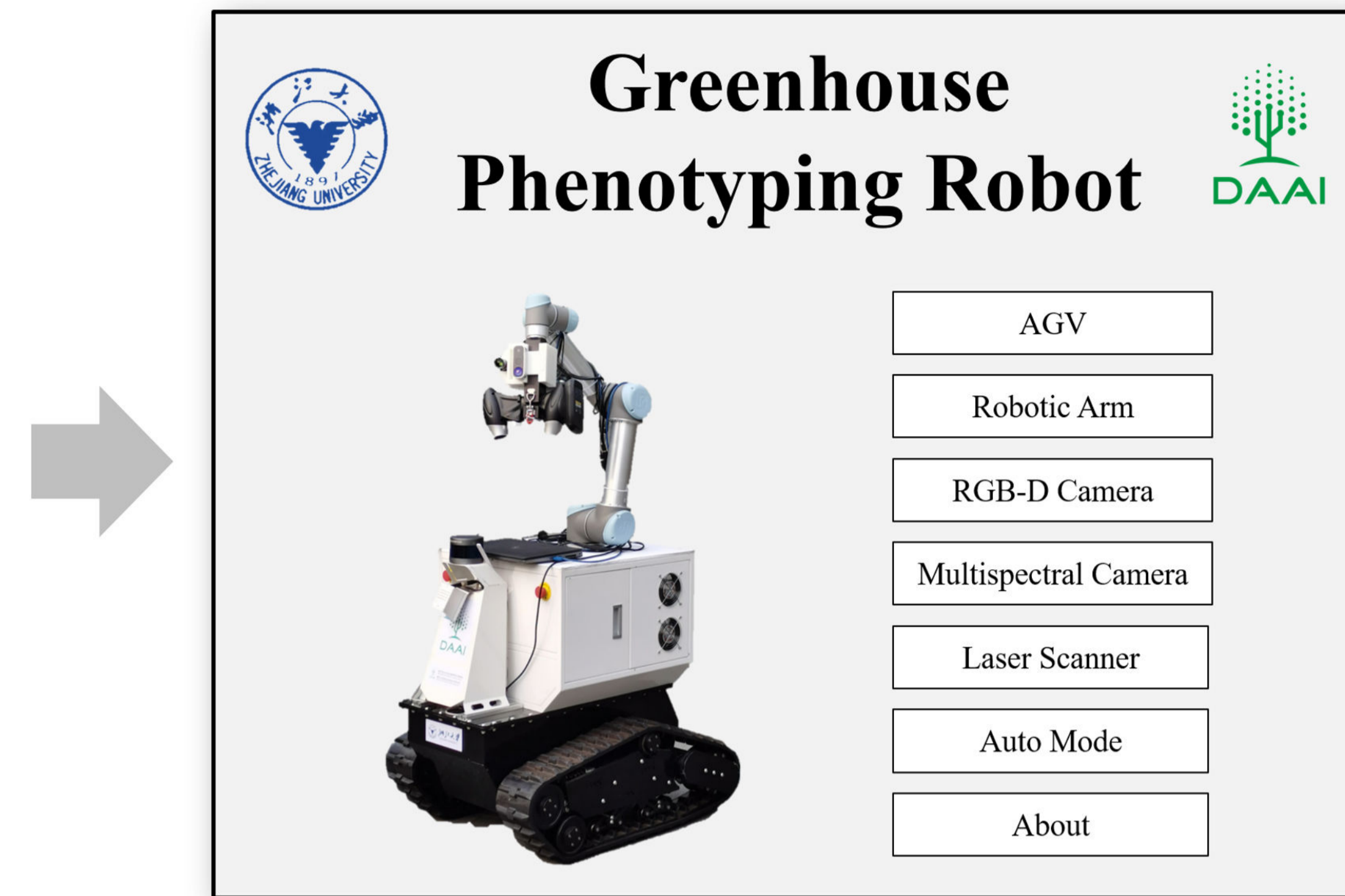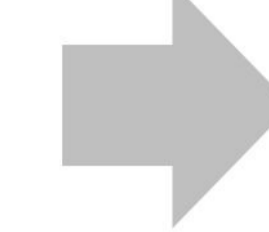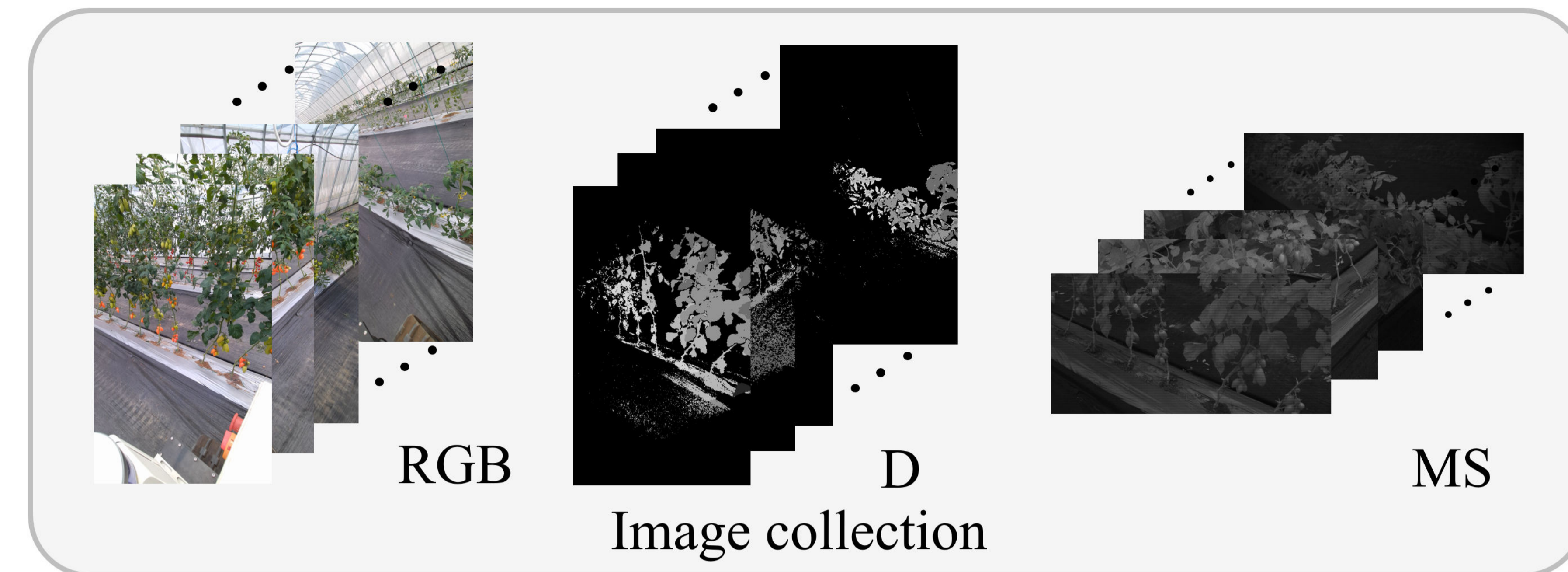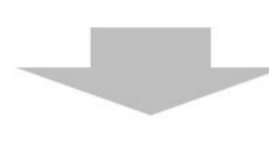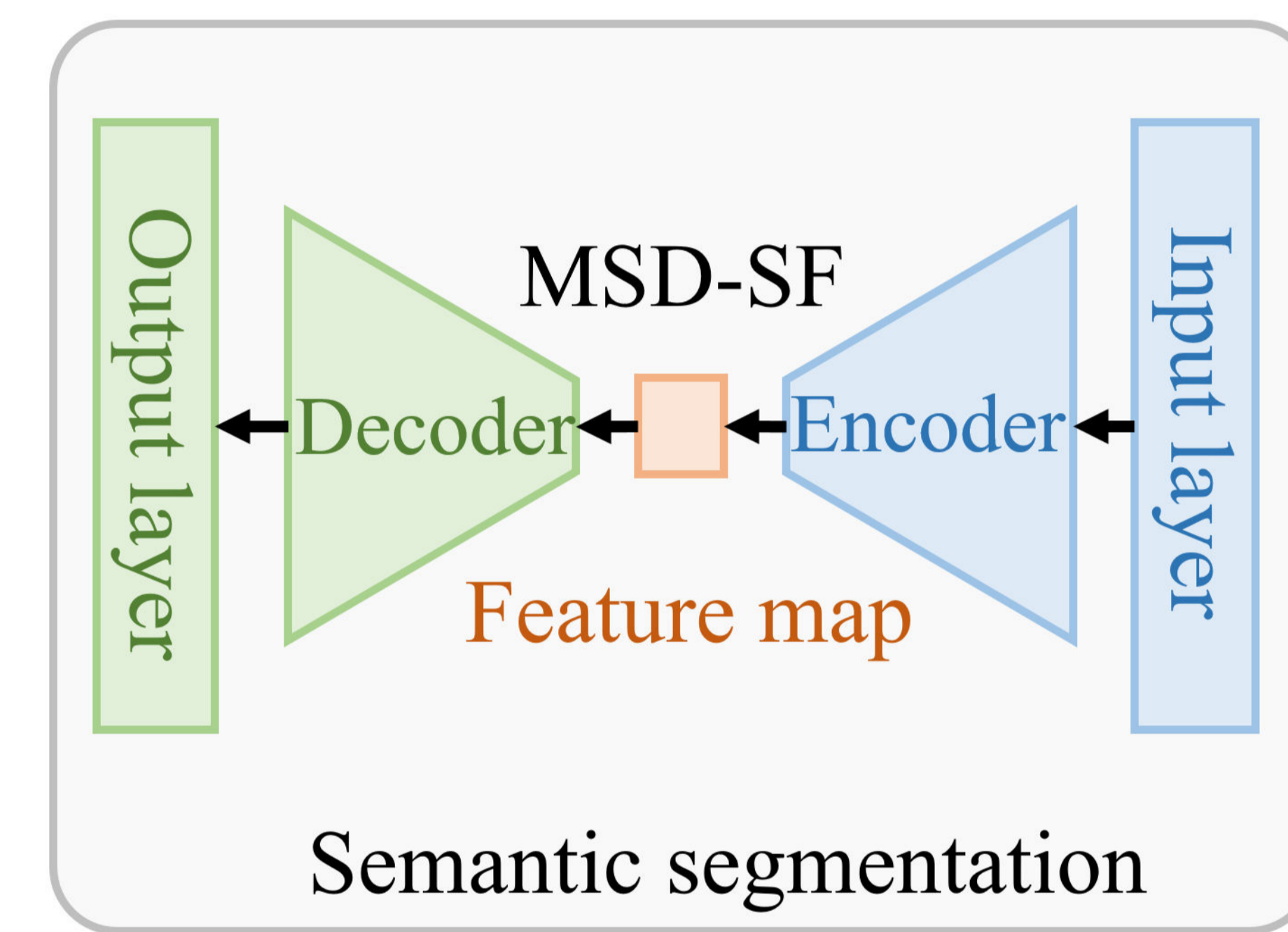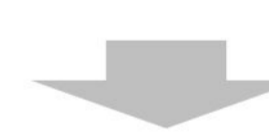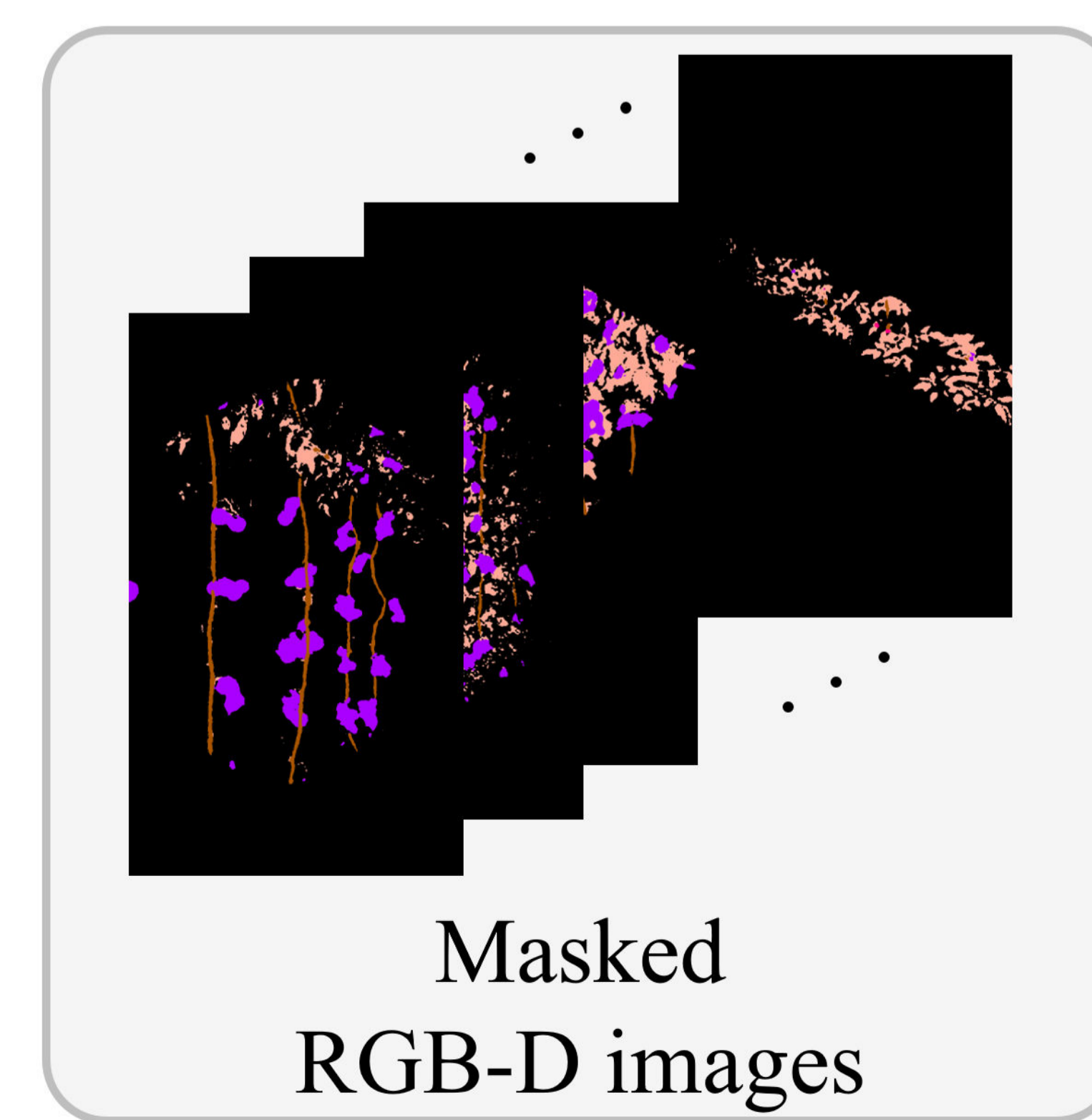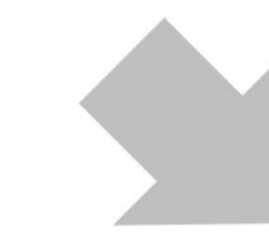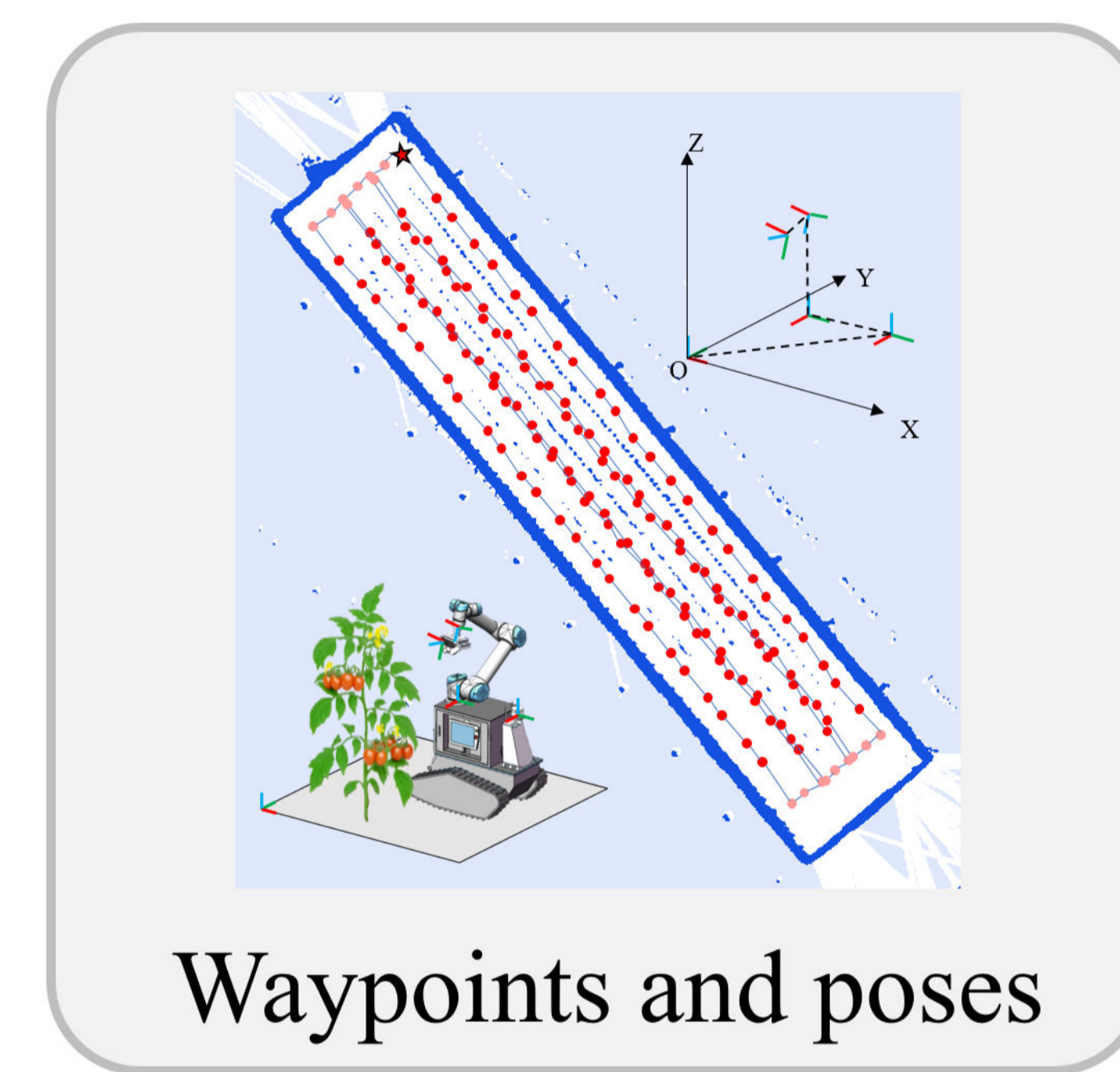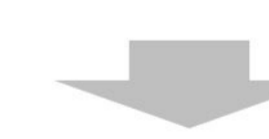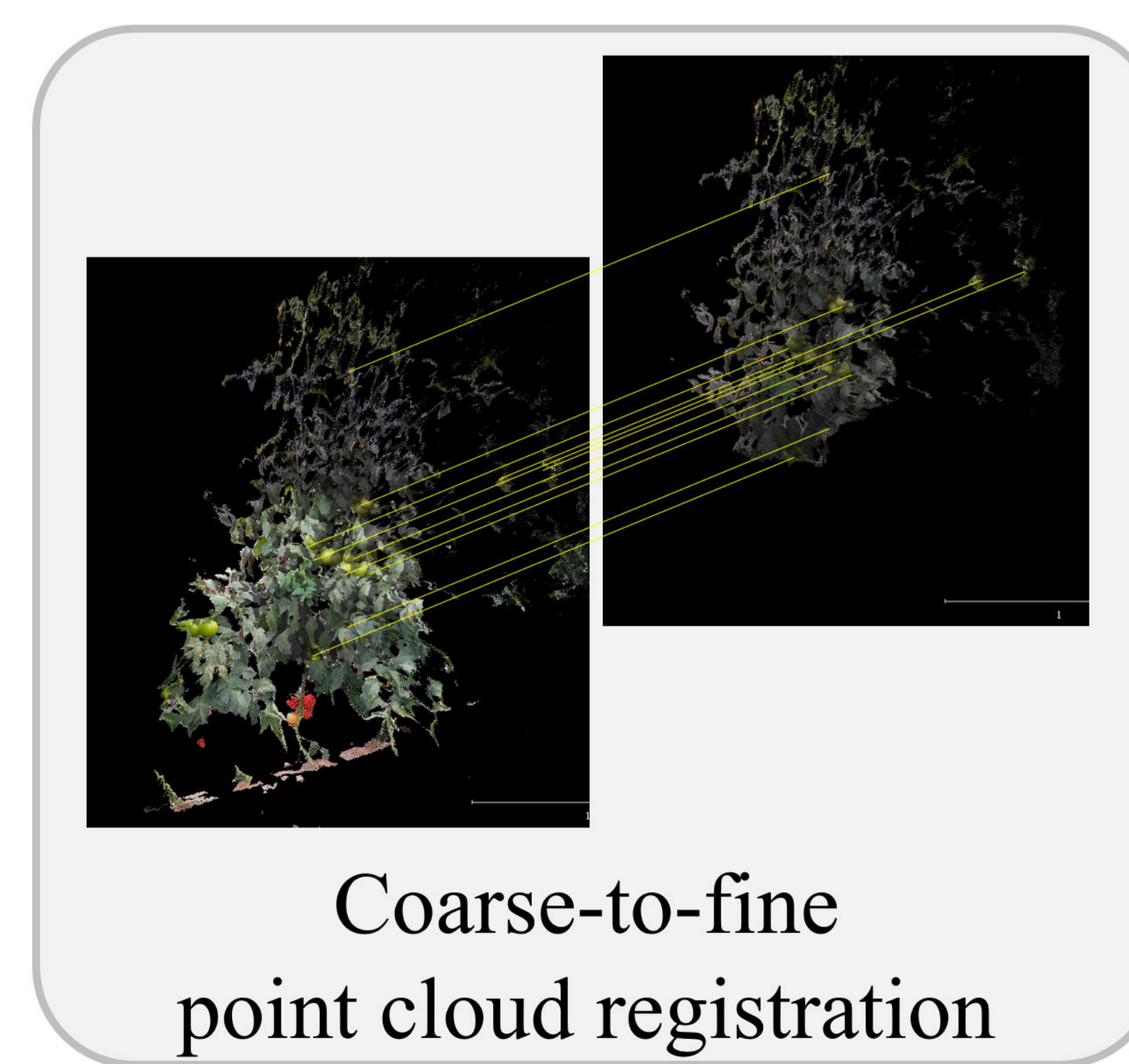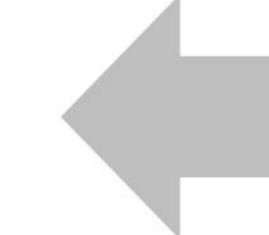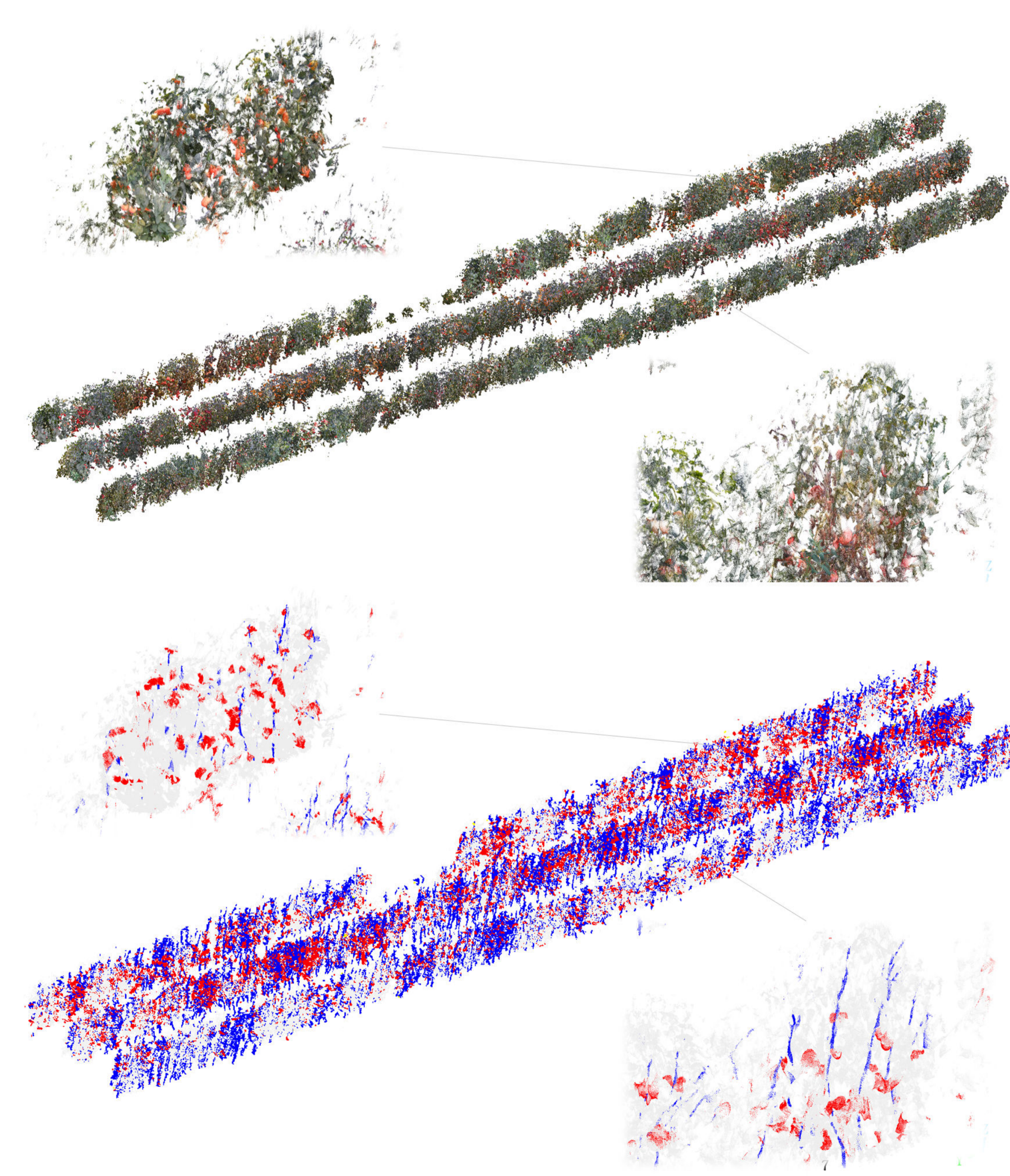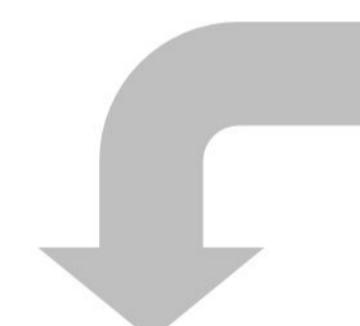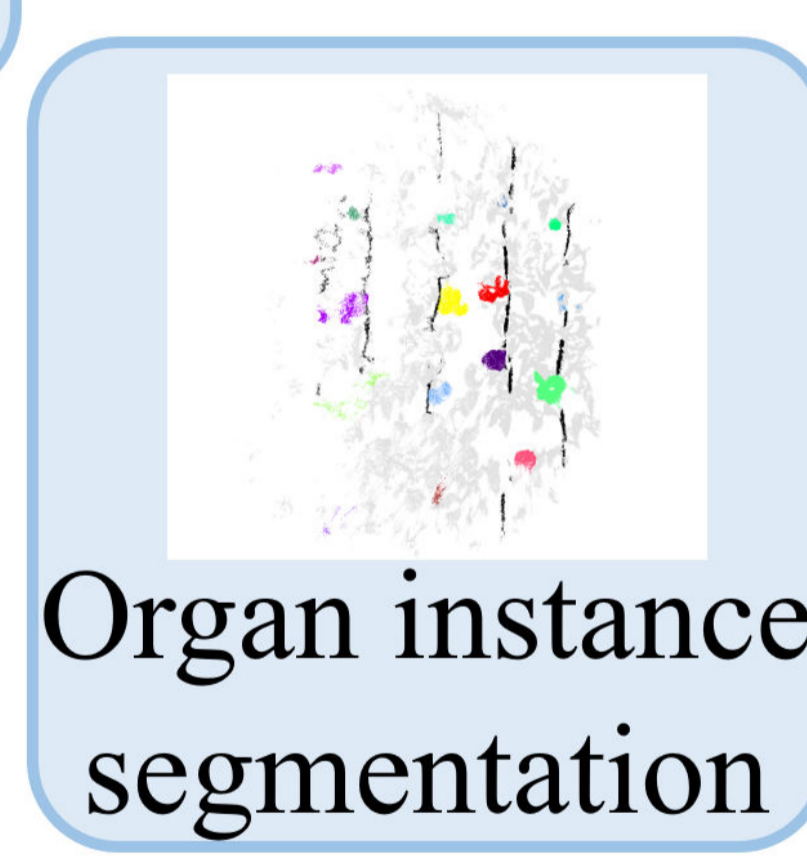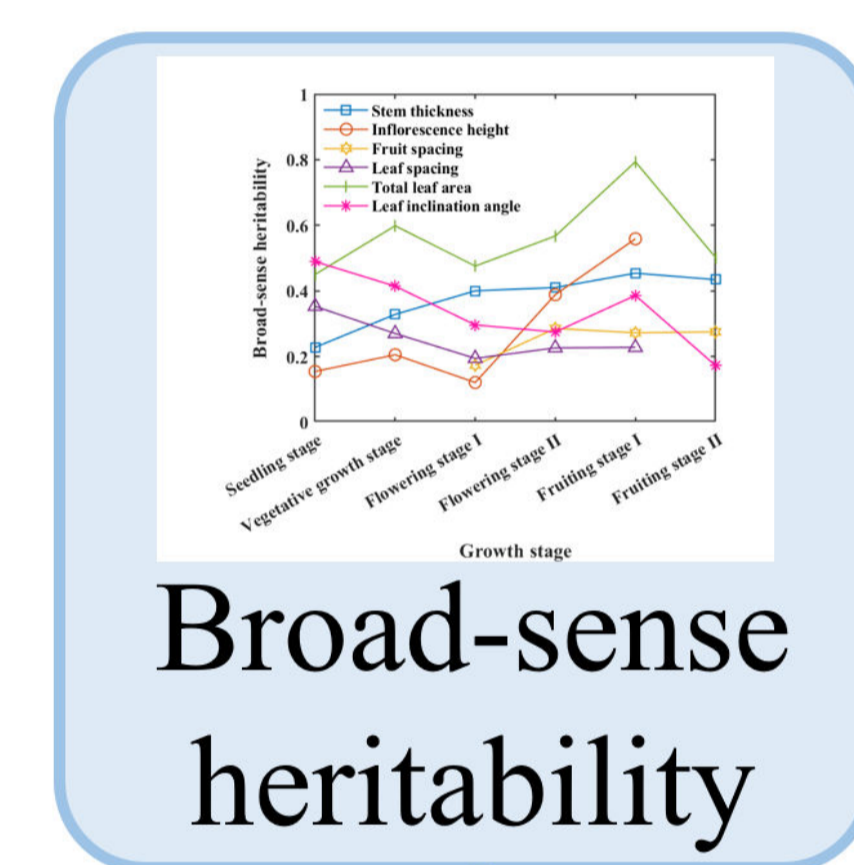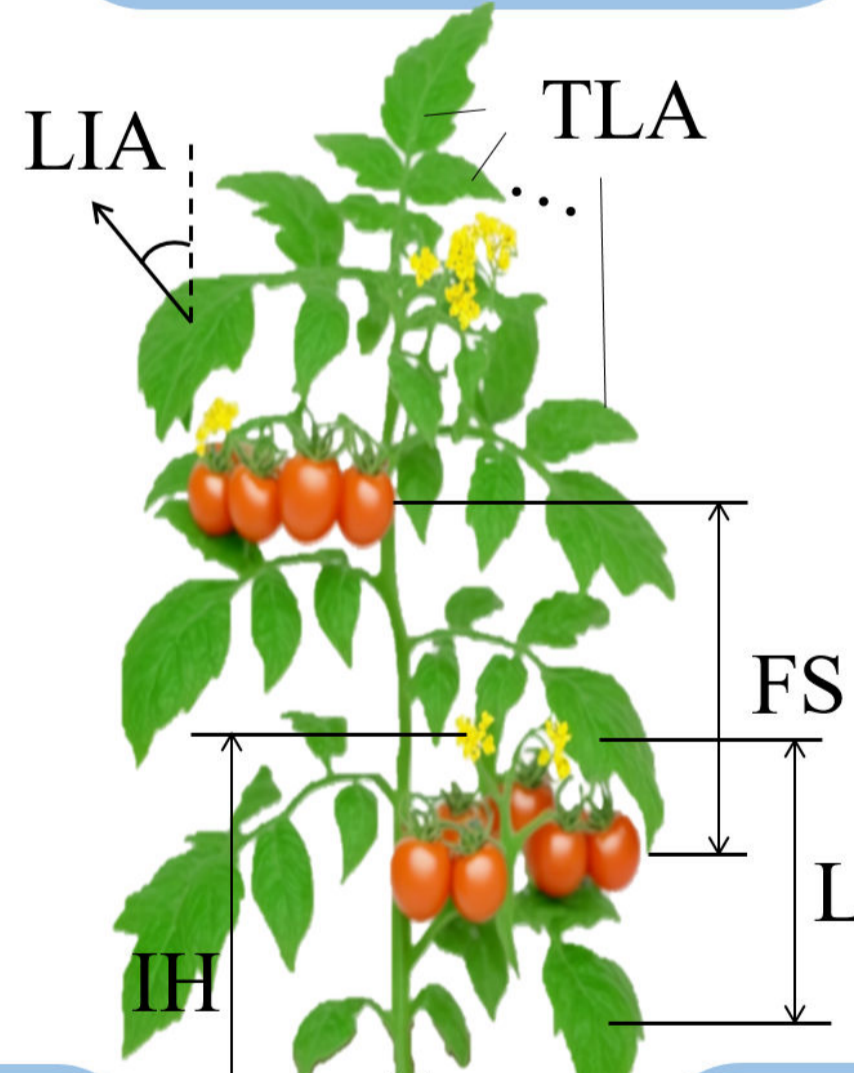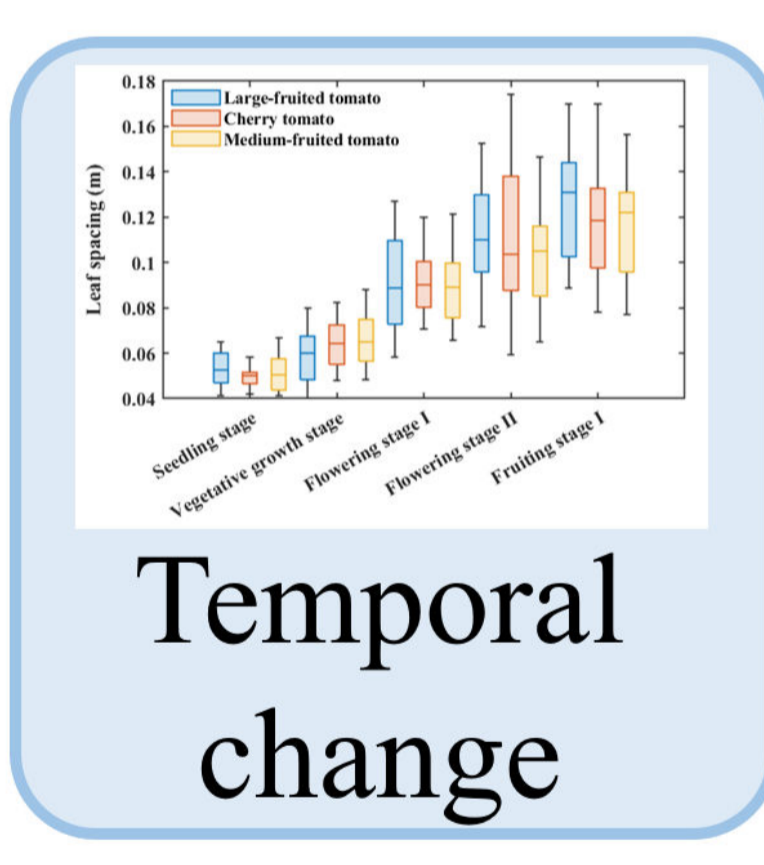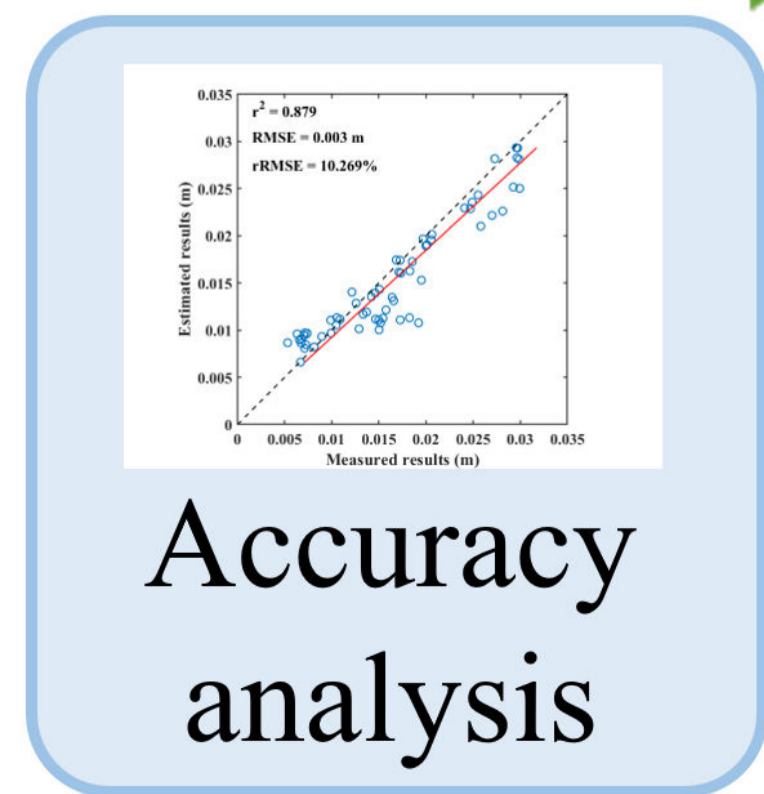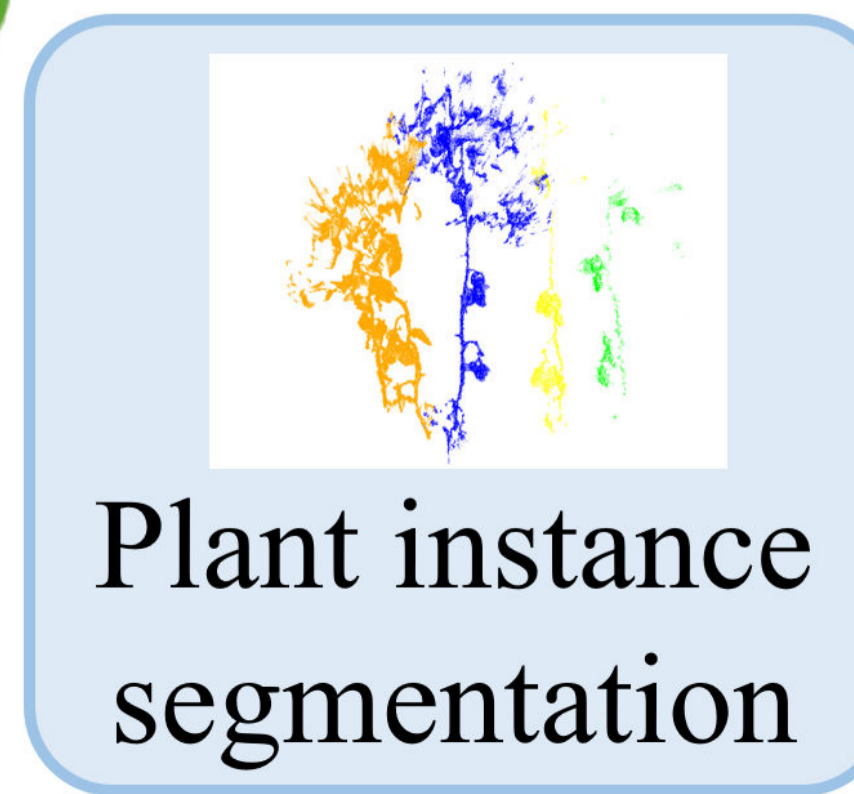

Supplement: Web_Material_uhaf109 [file web_material_uhaf109.zip › S1.pdf]

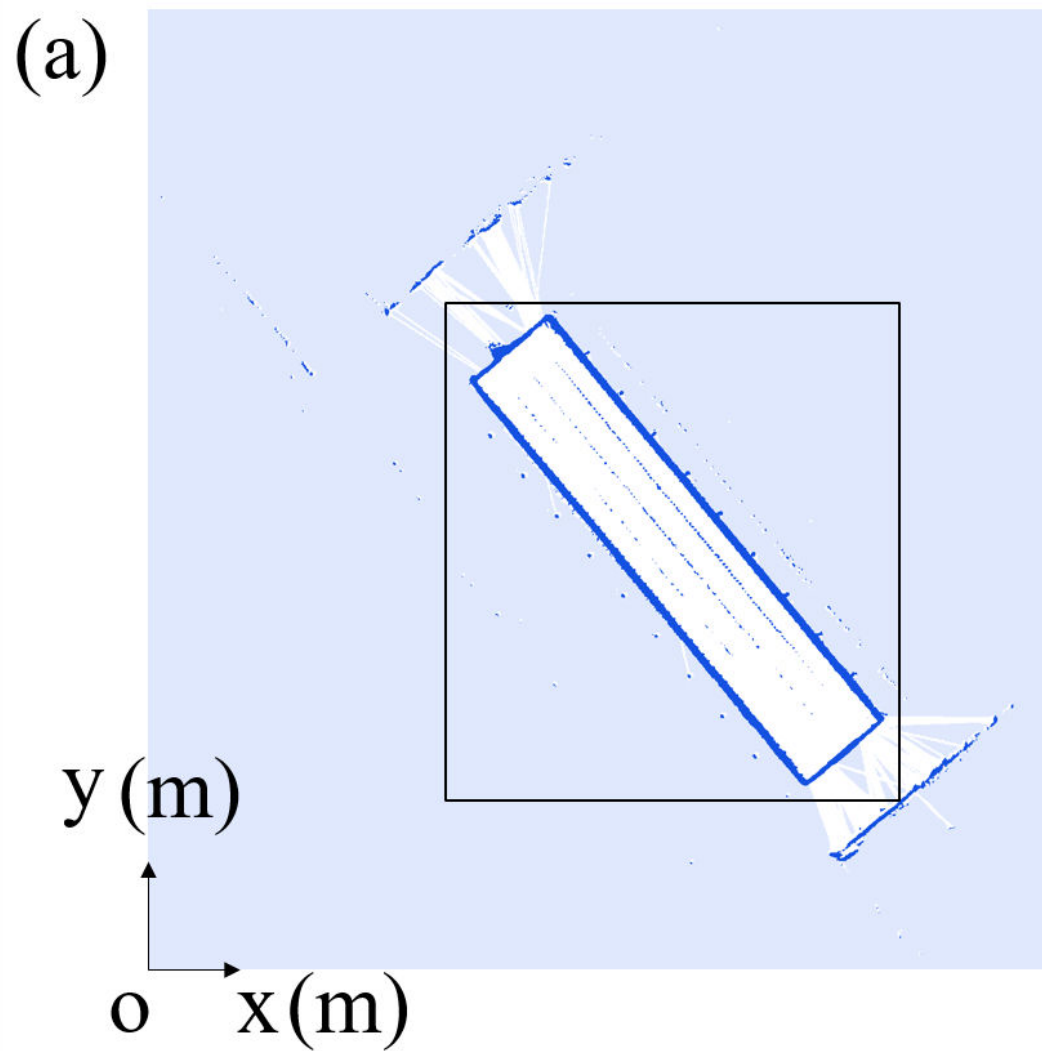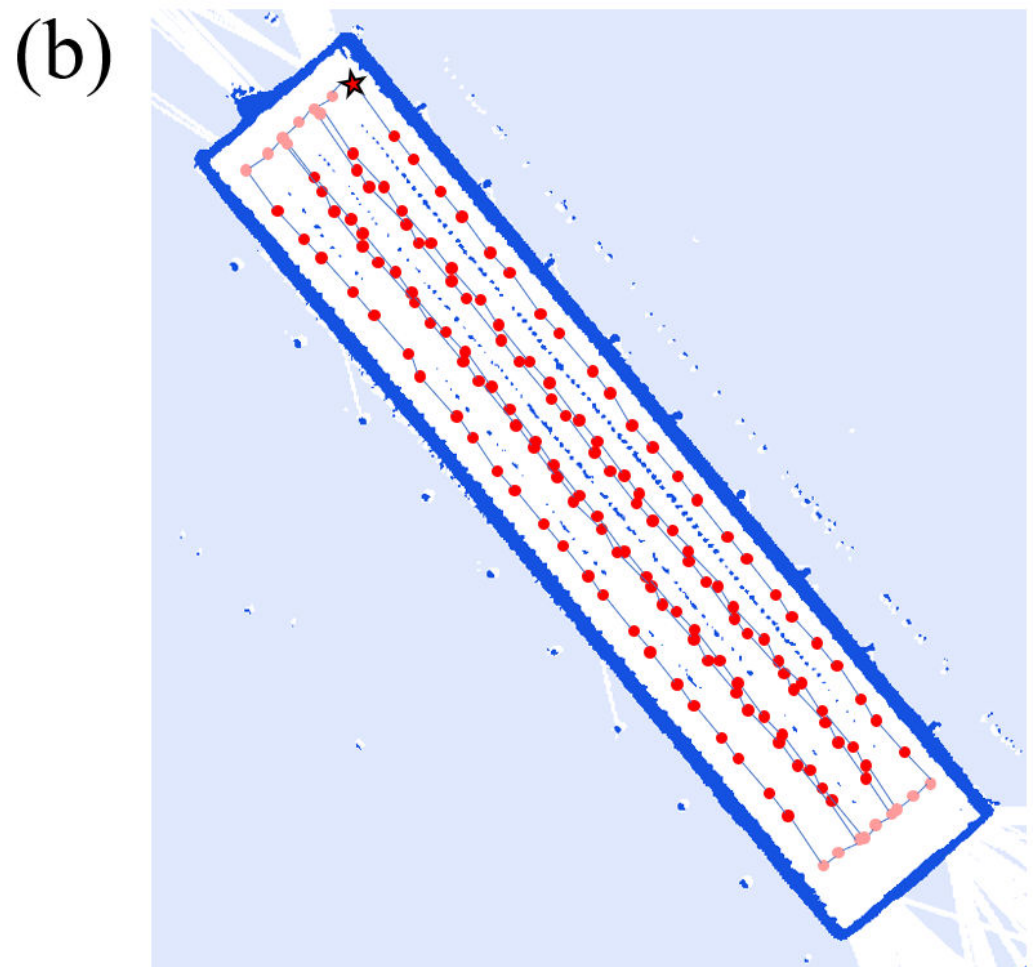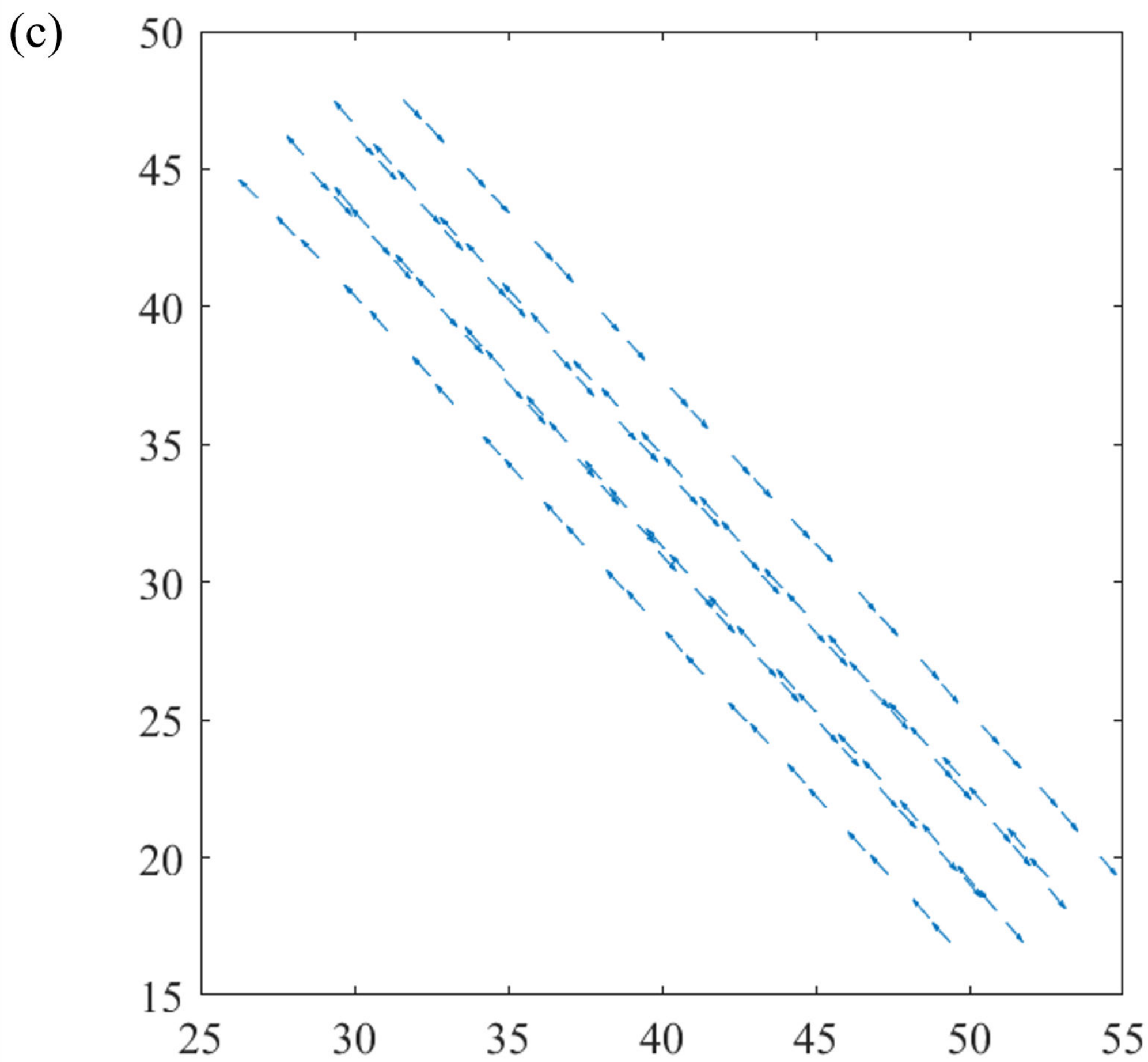

Supplement: Web_Material_uhaf109 [file web_material_uhaf109.zip › S2.pdf]

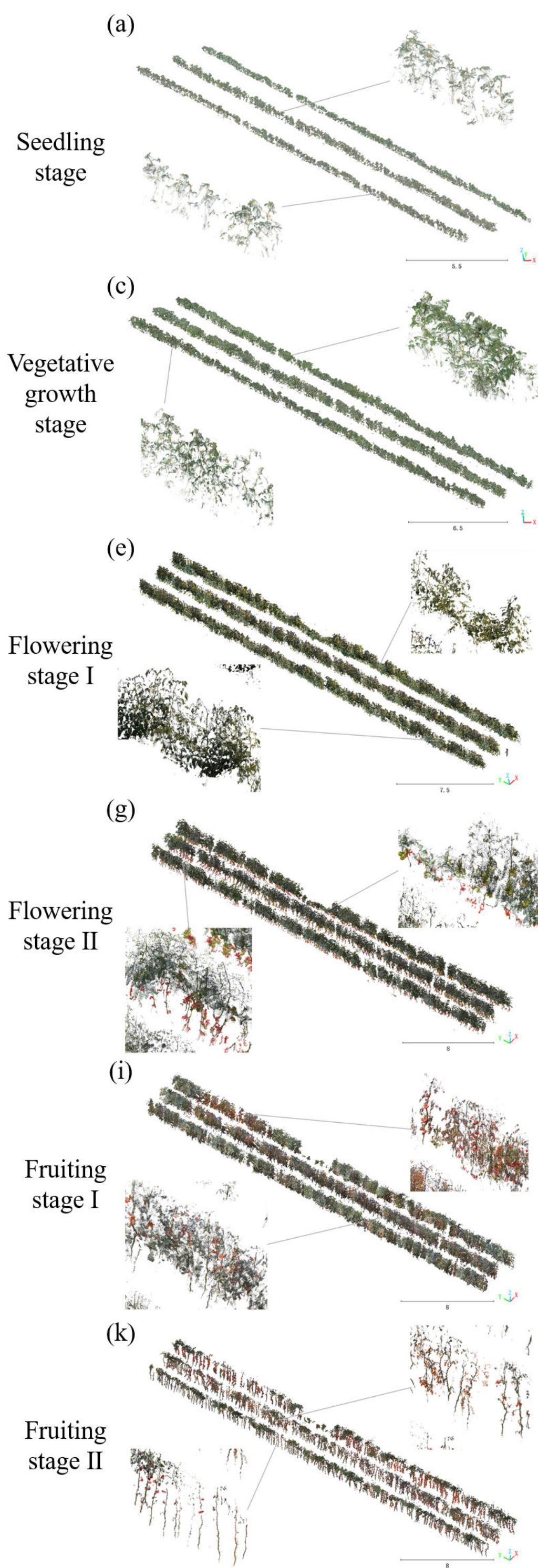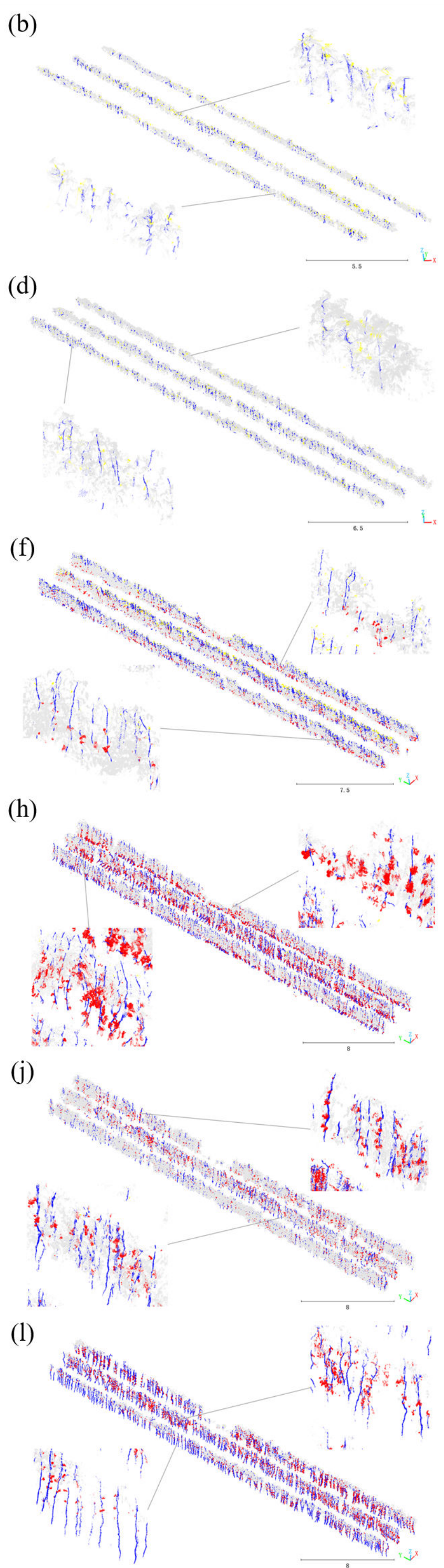

Supplement: Web_Material_uhaf109 [file web_material_uhaf109.zip › S3.pdf]

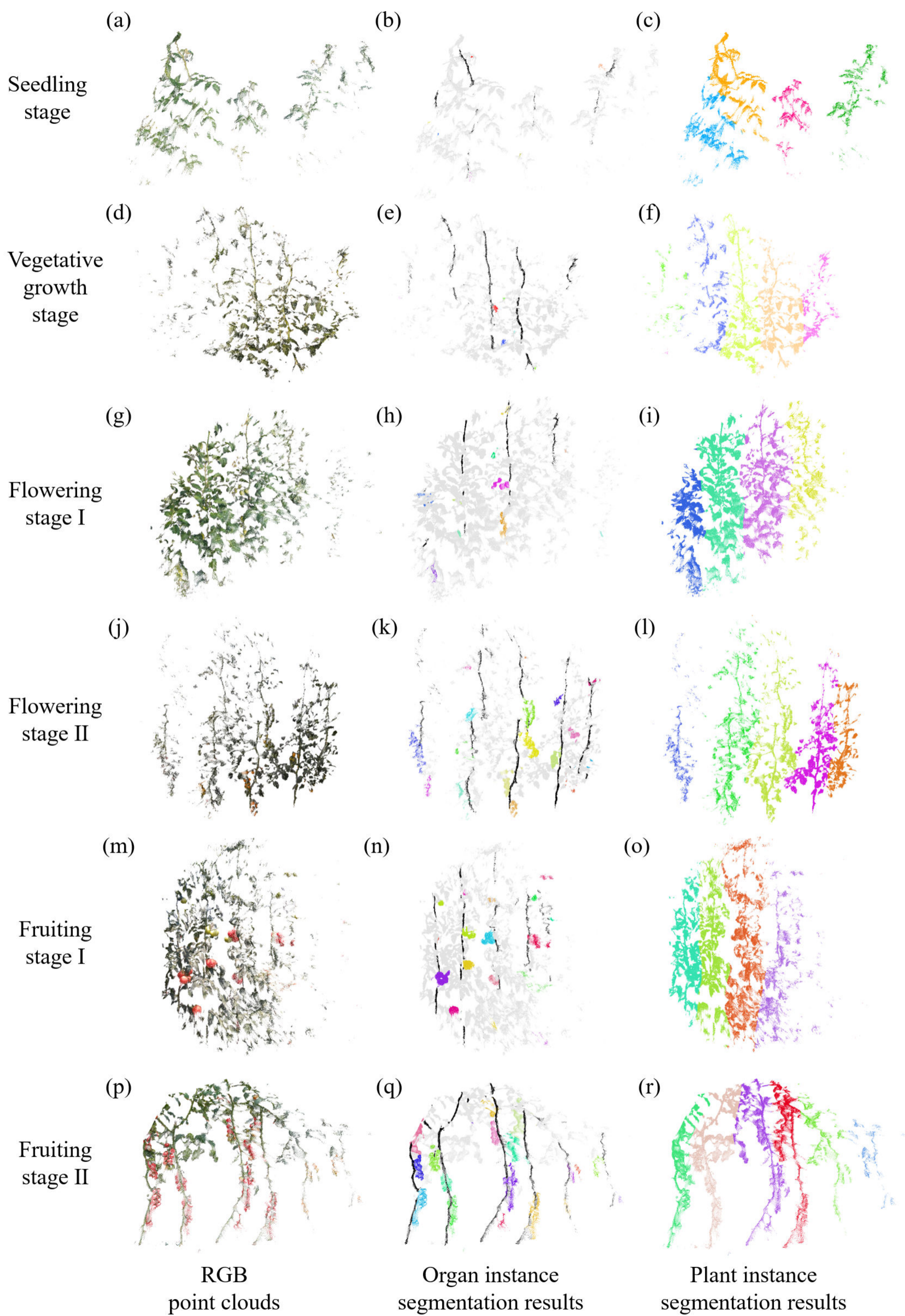

Supplement: Web_Material_uhaf109 [file web_material_uhaf109.zip › S4.pdf]

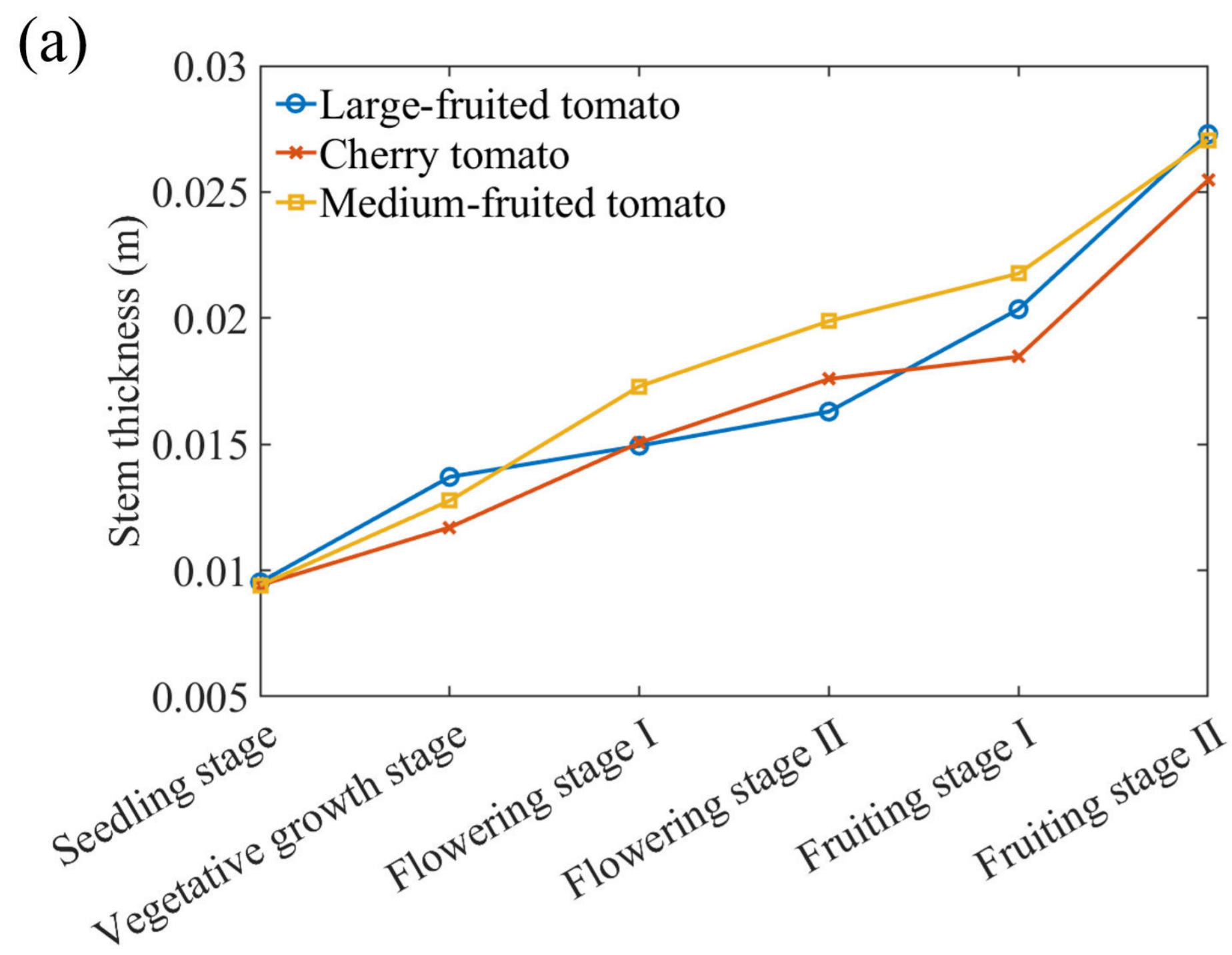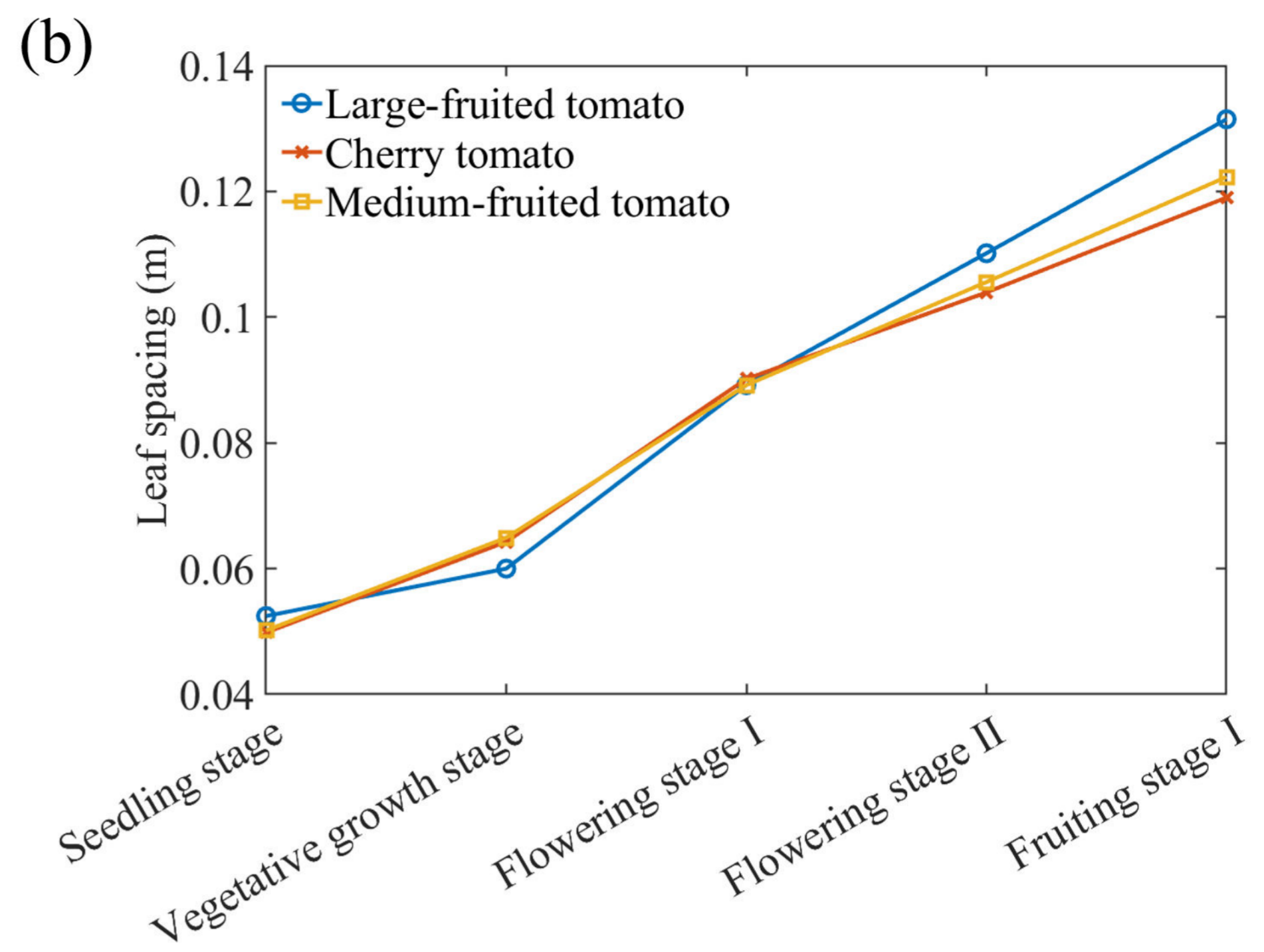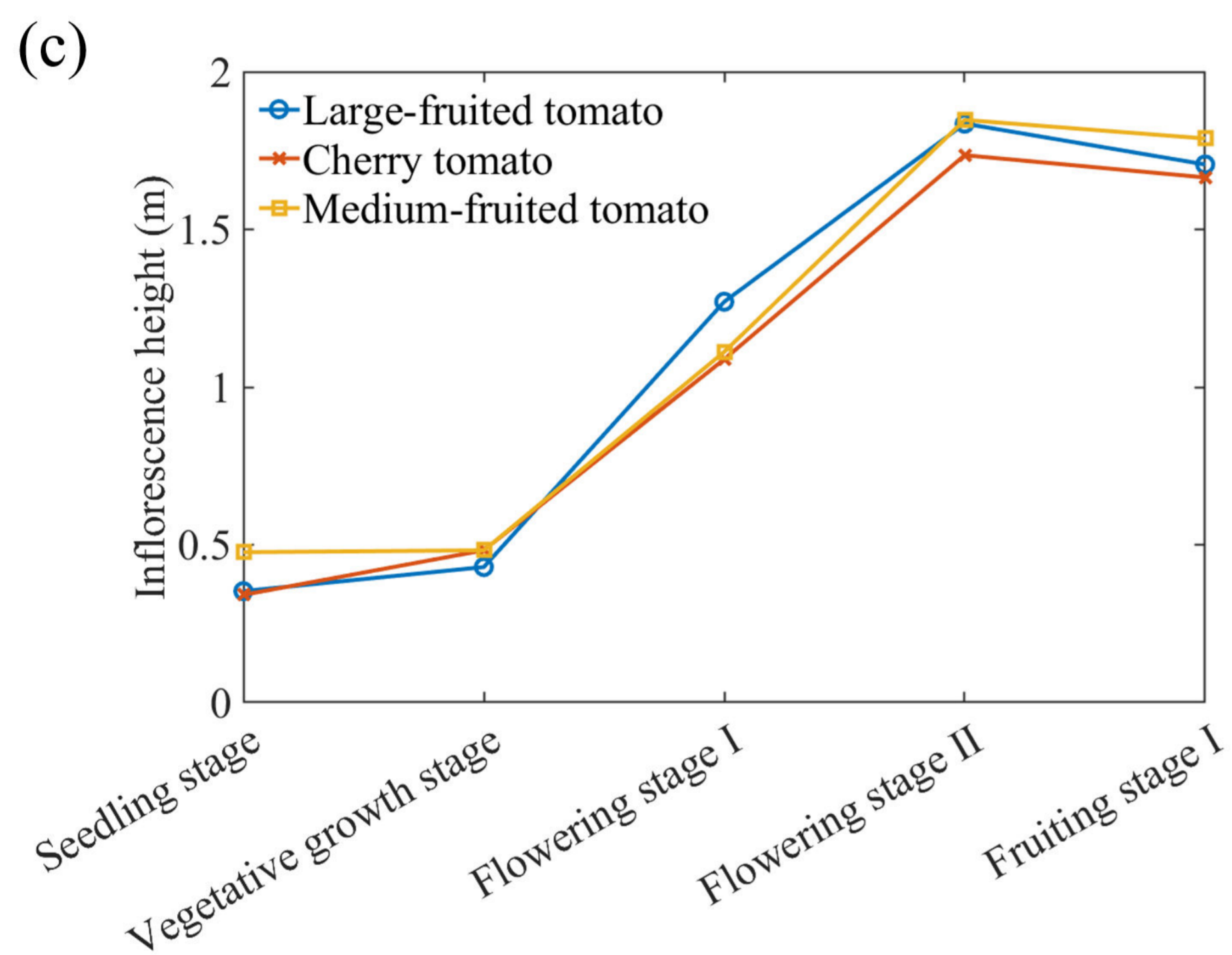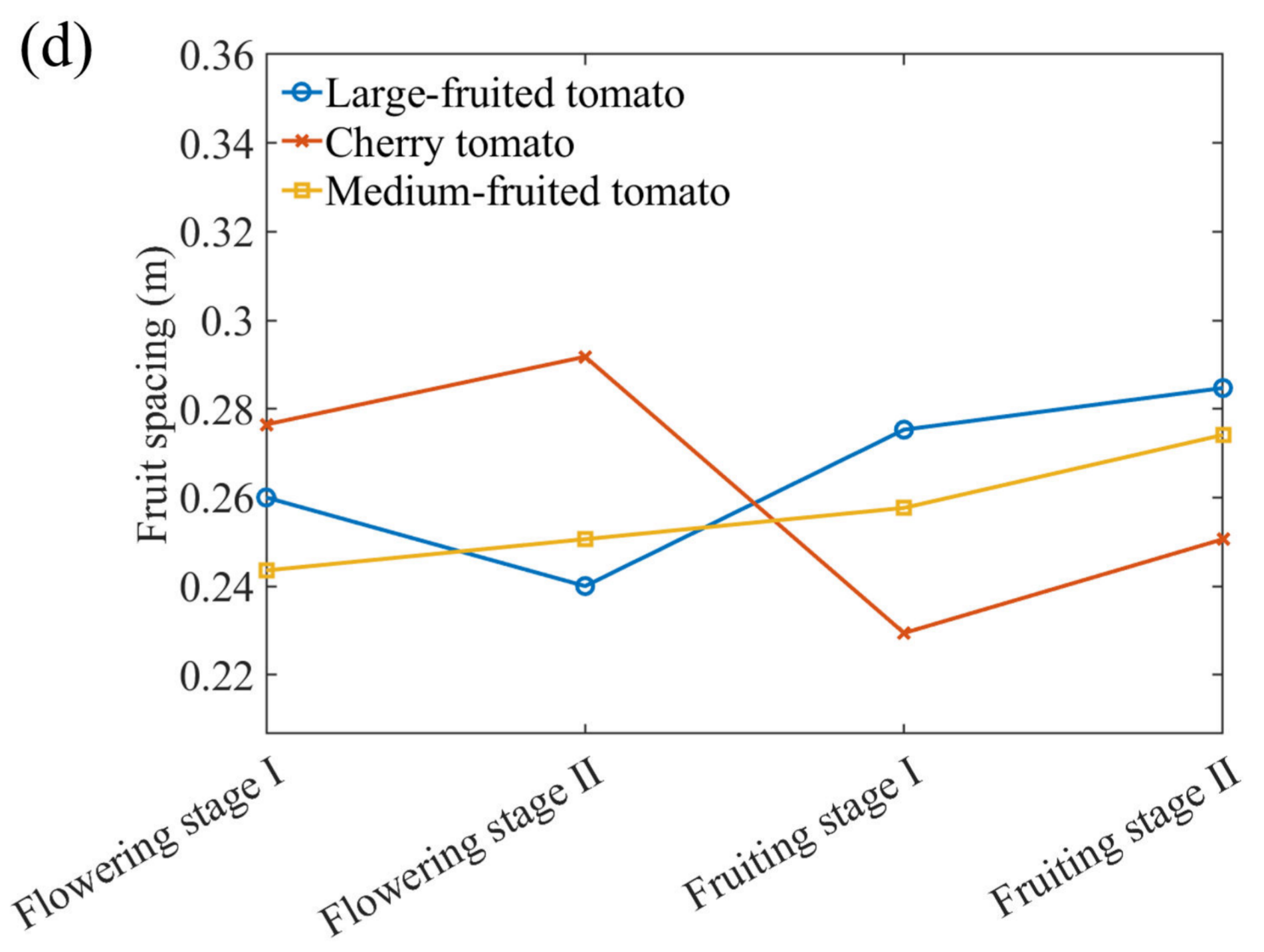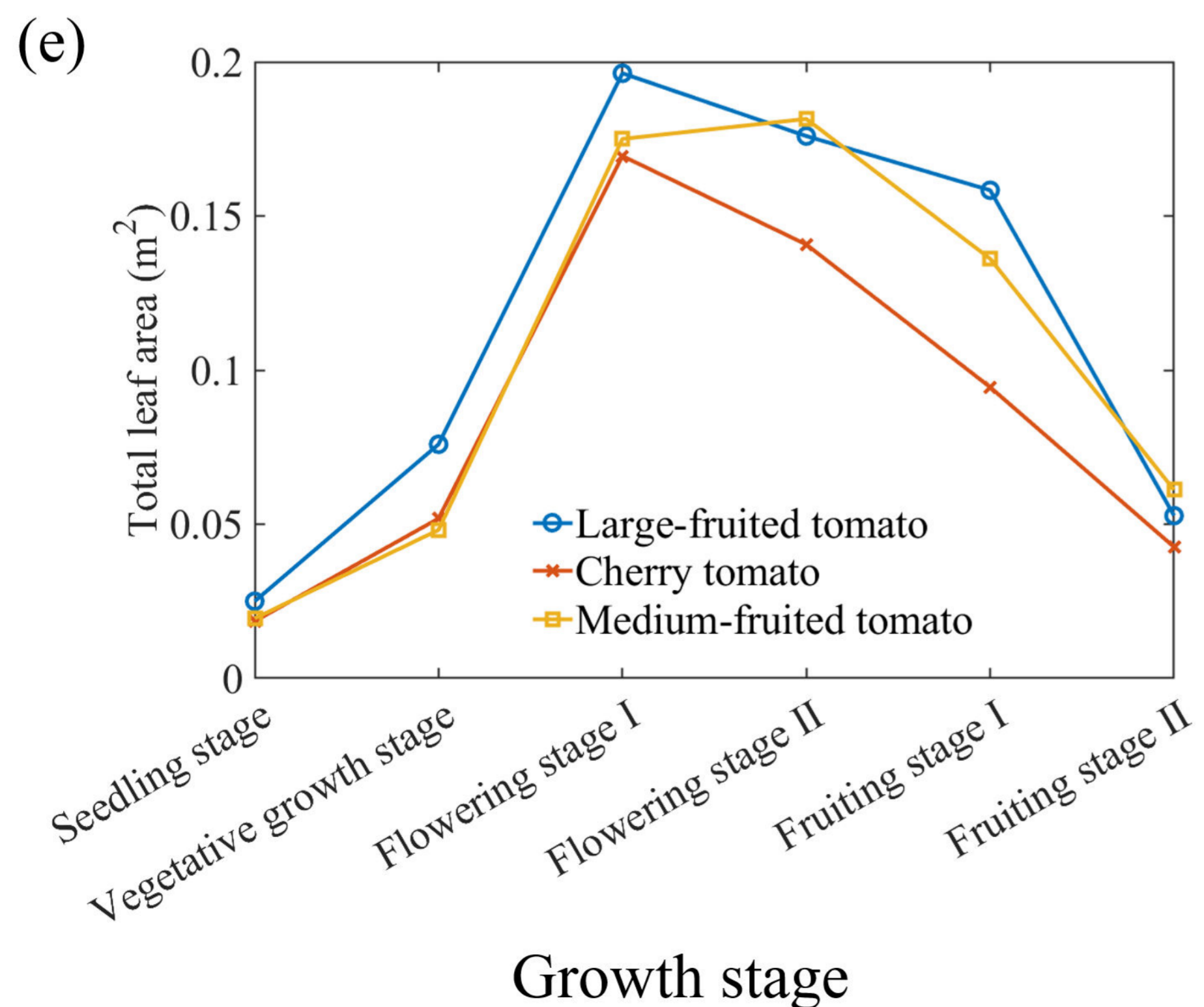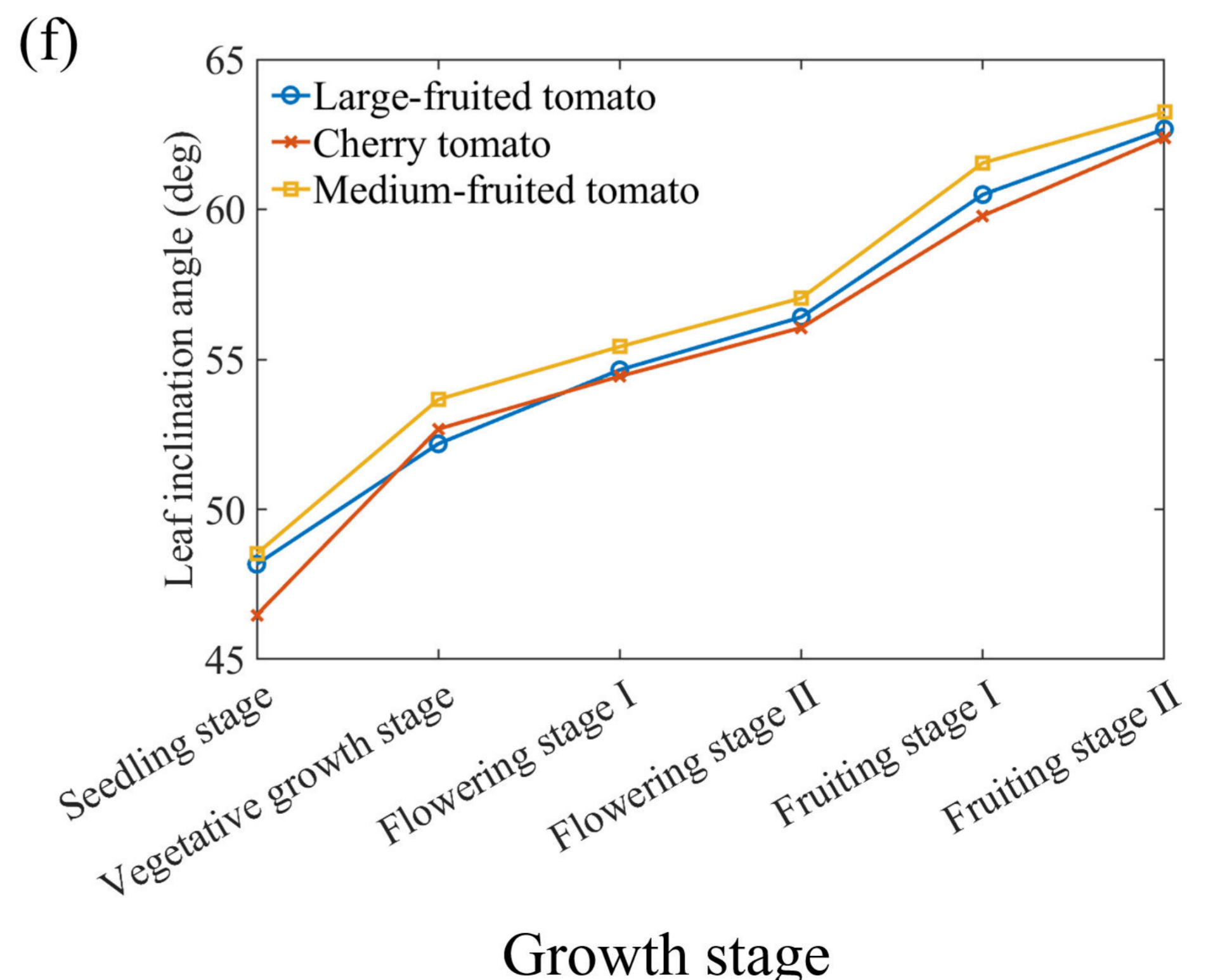

Supplement: Web_Material_uhaf109 [file web_material_uhaf109.zip › S5.pdf]

## (a) Route planning

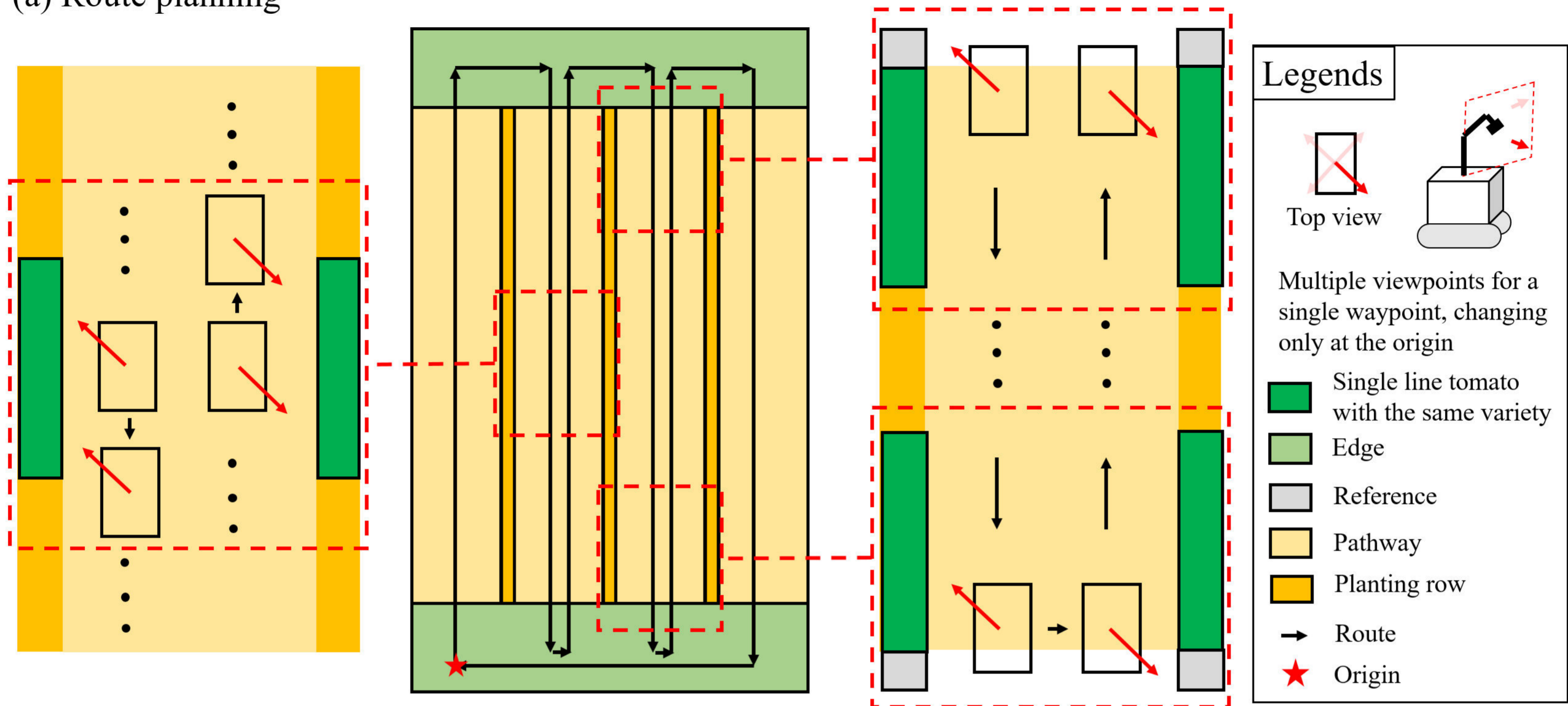

## (b) Data collection

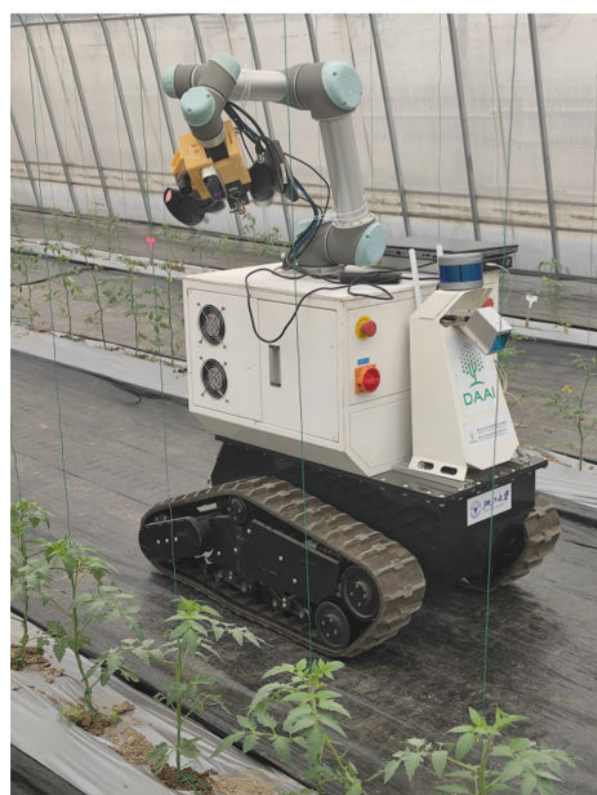

UGV

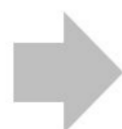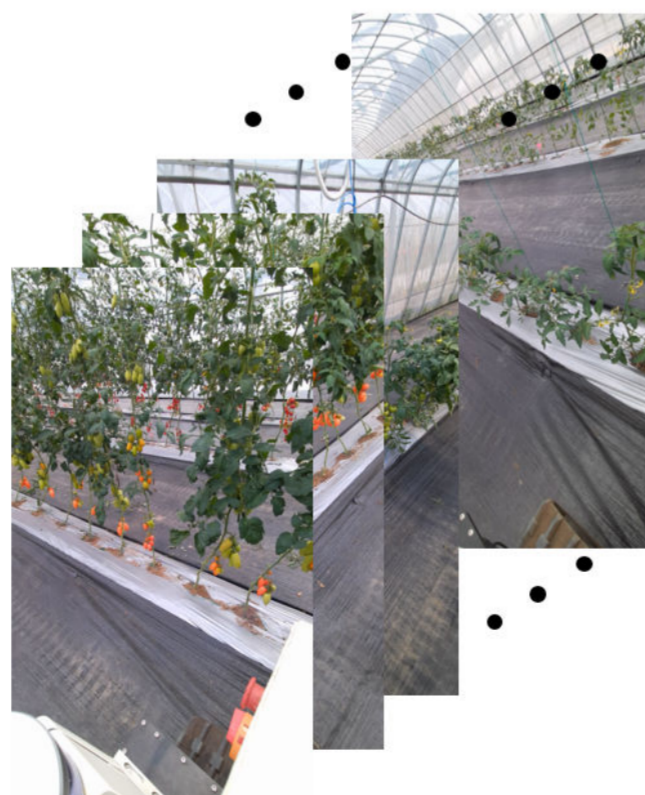

RGB images

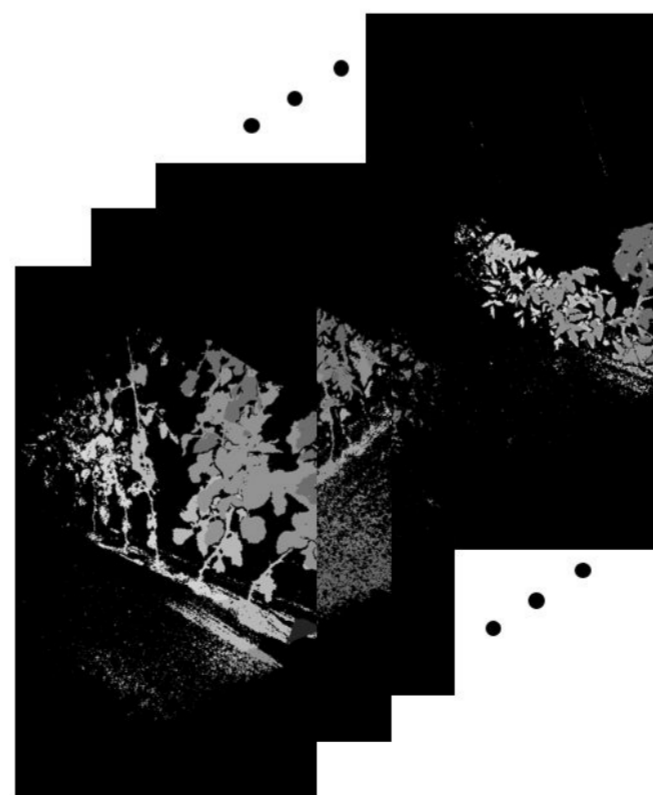

Depth images

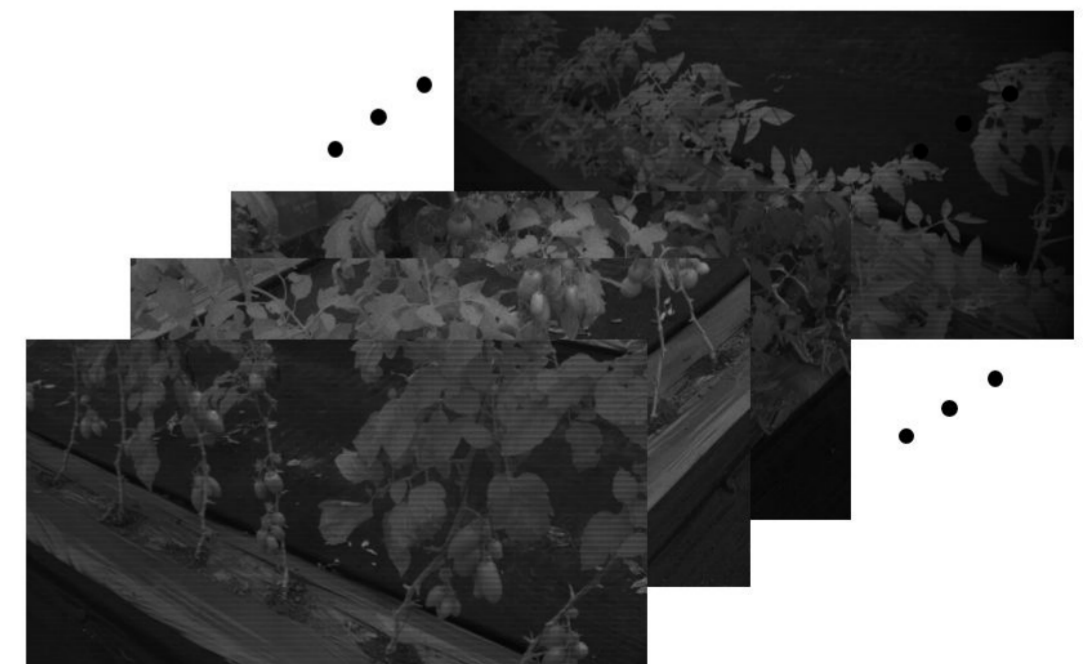

Multispectral images

Supplement: Web_Material_uhaf109 [file web_material_uhaf109.zip › S8.pdf]
